# Suppression of FOXM1 activities and breast cancer growth in vitro and in vivo by a new class of compounds

**DOI:** 10.1038/s41523-019-0141-7

**Published:** 2019-11-29

**Authors:** Yvonne Ziegler, Mary J. Laws, Valeria Sanabria Guillen, Sung Hoon Kim, Parama Dey, Brandi P. Smith, Ping Gong, Noah Bindman, Yuechao Zhao, Kathryn Carlson, Mayuri A. Yasuda, Divya Singh, Zhong Li, Dorraya El-Ashry, Zeynep Madak-Erdogan, John A. Katzenellenbogen, Benita S. Katzenellenbogen

**Affiliations:** 1 Departments of Molecular and Integrative Physiology, Urbana, IL 61801 USA; 2 Chemistry, Urbana, IL 61801 USA; 3 Illinois Informatics Institute and Department of Food Science and Human Nutrition, Urbana, IL 61801 USA; 4 Metabolomics Center of the Roy J. Carver Biotechnology Center, University of Illinois at Urbana-Champaign, Urbana, IL 61801 USA; 5 Department of Laboratory Medicine and Pathology, University of Minnesota Medical School, Minneapolis, MN 55455 USA

**Keywords:** Breast cancer, Transcription

## Abstract

The transcription factor FOXM1 is upregulated and overexpressed in aggressive, therapy-resistant forms of hormone receptor-positive and triple negative breast cancers, and is associated with less good patient survival. FOXM1 signaling is also a key driver in many other cancers. Here, we identify a new class of compounds effective in suppressing FOXM1 activity in breast cancers, and displaying good potency for antitumor efficacy. The compounds bind directly to FOXM1 and alter its proteolytic sensitivity, reduce the cellular level of FOXM1 protein by a proteasome- dependent process, and suppress breast cancer cell proliferation and cell cycle progression and increase apoptosis. RNA-seq and gene set enrichment analyses indicate that the compounds decrease expression of FOXM1-regulated genes and suppress gene ontologies under FOXM1 regulation. Several compounds have favorable pharmacokinetic properties and show good tumor suppression in preclinical breast tumor models. These compounds may be suitable for further clinical evaluation in targeting aggressive breast cancers driven by FOXM1.

## Introduction

The transcription factor FOXM1 is overexpressed and amplified in many types of cancers and is a master regulator of cancer cell division, aggressiveness, and metastasis.^1–8^ FOXM1 activity promotes all of the hallmarks of cancer, stimulating cell proliferation, genome instability, angiogenesis, and suppressing cell senescence.^9,10^ FOXM1 action is also associated with resistance to endocrine therapies in estrogen receptor (ER)-positive breast cancers and with resistance to radiation and many chemotherapies in several subtypes of breast cancer, and as well in many other cancers.^6,11–15^ We have shown that FOXM1 increases the cancer stem cell population, drives proliferation, motility and invasiveness, and therapy resistance, and we found that knockdown of FOXM1 in breast cancer cells could restore sensitivity to endocrine therapy.^15^ Hence, targeting FOXM1 activity is of great importance and could benefit many patients whose tumors are being driven by FOXM1. To meet this clinical need, we have therefore focused on the development of compounds to suppress FOXM1 activities.

In our search for regulators of FOXM1 activity, we assayed various members of a local chemical library for their inhibition of breast cancer cell proliferation and expression of FOXM1-signature genes, and we obtained a number of initial hits that were then expanded to a family of 1,1-diarylethylene mono and diamines, and their corresponding methiodide salts. These compounds were studied further to verify their direct target engagement with FOXM1, and structural modifications were made to improve their cellular potency and efficacy, and their in vivo activity.

Here, we report on the FOXM1 inhibitory activity of these compounds in cell-free and cell-based assays, and in in vivo preclinical breast tumor models. The compounds bind directly to FOXM1 and affect FOXM1 stability, decrease the cellular level of FOXM1, effectively suppress proliferation and increase apoptosis of FOXM1-containing human breast cancer cells, and block the expression of FOXM1-regulated genes. One of the compounds had good oral efficacy in suppressing the growth of FOXM1-containing breast tumors in NOD-SCID-gamma (NSG) mice, and several others had good efficacy in tumor suppression by subcutaneous administration. Our findings identify and characterize a new class of compounds that effectively antagonize FOXM1 actions and tumor growth, and may be suitable for further clinical evaluation in targeting aggressive breast cancers driven by FOXM1.

## Results

### Effects of compounds on proliferation of a panel of breast cells

**Fig. 1:**
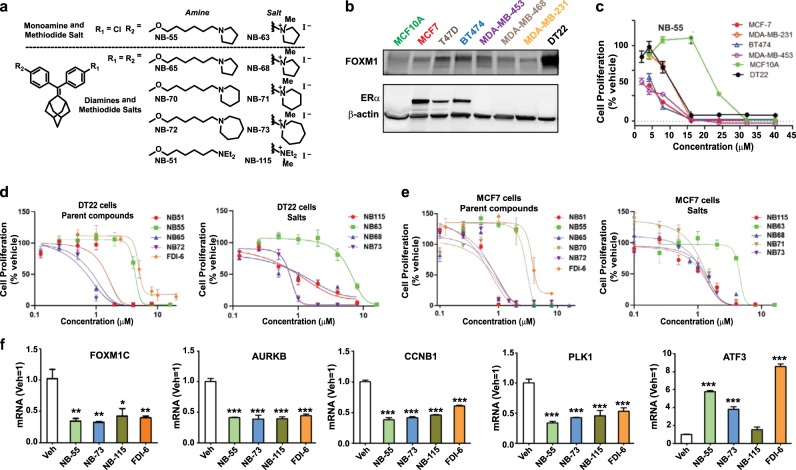
Compounds studied and effects of the compounds on inhibition of cell proliferation and regulation of FOXM1 target gene expression. **a** Structures of the 1,1-diarylethylene monoamine, diamines, and their methiodide salts we have studied. **b** Western blot analysis shows that the cell lines differ in their relative content of FOXM1 protein and in the expression of ERα. **c** Inhibition of cell proliferation by **NB-55** examined in dose-response studies in these cell lines. Values are mean ± SD with assays done in triplicate. **d, e** Inhibition of cell proliferation by parent amine and their methiodide salt compounds in DT22 or MCF7 cells incubated for 3 days with the indicated concentrations of each compound or with **FDI-6** for comparison. Assays were run in triplicate. Values are mean ± SEM. **f** Inhibition of FOXM1 target gene expression by parent amine and methiodide salt compounds. Inhibition of the expression of FOXM1 upregulated genes (FOXM1C, AURKB, CCNB1, PLK1) and reversal of FOXM1 downregulation of ATF3 in MCF7 cells. Cells were incubated for 24 h with each compound at their IC_50_ concentration based on cell proliferation assays. RNA was extracted from cells and expression of different genes was monitored by qRT-PCR. Assays were run in triplicate. Values are mean ± SEM. *p < 0.05; **p < 0.01; ***p < 0.001.

The 1,1-diarylethylene compounds we have studied are shown in Fig. 1a*.* They were obtained after FOXM1 target engagement verification and structural optimization of initial hits from a local chemical library that were identified through cell-based assays of inhibition of breast cancer cell proliferation and FOXM1-regulated gene expression, described below. The set of compounds is composed of one monoamine and four diamines, in each case with the corresponding methiodide salt that was used to optimize their in vivo properties.

We used a panel of human breast cancer cell lines and the non-tumorigenic MCF10A breast cell line that differed in their FOXM1 protein content (high to intermediate levels, DT22, MCF7, T47D, BT474, MDA-MB-453, MDA-MB-468, and MDA-MB-231 cells; and low level, MCF10A cells) to examine the effects of potential FOXM1 inhibitor compounds on cell proliferation. All cell lines are ER-negative except for MCF7, T47D, and BT474 cells (Fig. 1b). As seen in Fig. 1c, where we monitored the effects of the earliest lead compound, the monoamine **NB-55**, we found that cells with high or intermediate levels of FOXM1 protein showed relatively similar dose-responses for inhibition of cell proliferation, whereas MCF10A, with a low level of FOXM1, showed a reduced sensitivity to **NB-55**, requiring a concentration of **NB-55** five times higher to achieve equal 50% suppression of proliferation (Fig. 1c).

Because the IC_50_ for inhibition of proliferation by the monoamine **NB-55** was only ca. 2–10 µM in the breast cancer cell lines, we compared **NB-55** with some members of the diamine series (**NB-51, 55, 65, 70, 72**), as well as with their methiodide salts (**NB-63, 68, 71, 73, 115**), for possible improved potency. In these studies, we also used the FOXM1 inhibitor **FDI-6**^16^ as a comparator compound (Fig. 1d, e). Notably, all of the diamines were markedly more potent than the monoamine **NB-55** and also **FDI-6** in suppressing cell proliferation in both ER-negative DT22 cells and ER-positive MCF7 cells. In these cell assays, the methiodide salts, whether of the monoamine (**NB-63**) or the diamines (**NB-68, 71, 73, 115**), had potencies very similar to those of their parent amine compounds. Dose-response antiproliferative studies in additional cell lines beyond the ER-positive MCF7 and the basal/claudin low triple negative DT22 cells gave similar findings. Thus, in triple negative MDA-MB-231 and MDA-MB-453 breast cancer cells, IC_50_ values for the monoamine **NB-55** were 3–5 µM, whereas the diamines and their methiodide salts gave IC_50_ values of 0.6 ± 0.14 µM, very similar to those observed with MCF7 and DT22 cells. Of interest, the diamine salt tested, **NB-73**, also effectively suppressed the proliferation of tamoxifen-resistant MCF7 breast cancer cells, whose growth was weakly stimulated rather than suppressed by trans-hydroxytamoxifen (Supplementary Fig. 1).^17^

Analysis of gene expression using **NB-55**, **NB-73**, **NB-115**, and **FDI-6** for comparison, at their IC_50_ concentration for suppression of cell proliferation, showed that they significantly reduced the expression of classic FOXM1 stimulated target genes^15,18,19^ (FOXM1C, AURKB, CCNB1, and PLK1) and increased expression of ATF3, a gene suppressed by FOXM1 (Fig. 1f).

### Direct binding of compounds to FOXM1 and increased FOXM1 sensitivity to proteolysis upon compound binding

**Fig. 2:**
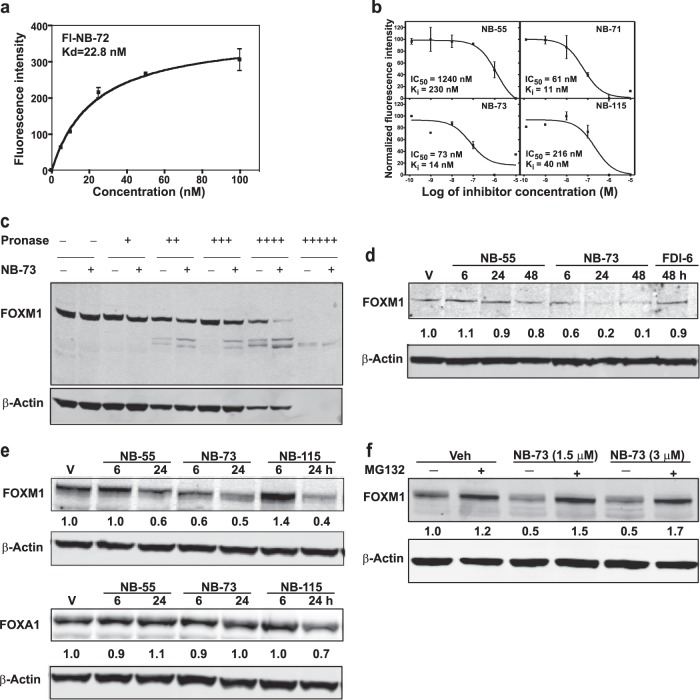
Fluorescence assays of binding of compounds to FOXM1 protein. **a** Direct binding of **Fl-NB-72** to FOXM1 by tr-FRET (*n* = 2, error bars ± SD). **b** Representative competitive binding assays of compounds using **Fl-NB-72** as the tr-FRET probe (*n* = 2, error bars ± SD). **c** DARTS assay showing increased susceptibility of FOXM1 protein to degradation by pronase upon exposure of cell extracts to **NB-73**. β-Actin in cell extracts is also shown for comparison. **d** Effect of treatment of MDA-MB-231 cells with **NB-55** (5 µM), **NB-73** (1.5 µM), or **FDI-6** (8 µM) on FOXM1 level or **e** treatment of MCF7 cells with **NB-55** (8 µM), **NB-73** (4 µM), **NB-115** (4 µM) on the cellular level of FOXM1 or FOXA1 protein (a different forkhead protein for comparison) monitored by Western blot over time. **f** Reversal of the downregulation of FOXM1 by cotreatment of MDA-MB-231 cells with **NB-73** (1.5 or 3 µM) and the proteasome inhibitor MG132 (1 µM) for 16 h. Western blots of cell extracts are shown. Numbers shown in panels **d–f** are values for FOXM1 or FOXA1 corrected for β-Actin in each sample.

We used two approaches to investigate whether these antiproliferative compounds were engaging FOXM1 directly. First, we developed a time-resolved fluorescence resonance energy transfer (tr-FRET) assay for direct FOXM1 binding. We adapted the diamine compound **NB-72** to create an acceptor fluorophore **Fl-NB-72** by replacing one amine appendage with fluorescein, and we attached a terbium donor to purified, full length FOXM1 through a streptavidin-biotin linker. FOXM1 titrations showed that **Fl-NB-72** bound to the FOXM1 protein with high affinity (K_d_ = 23 nM) (Fig. 2a).

The FOXM1 interaction of the ten compounds we had studied were assessed by a competition assay, monitoring the decrease in FRET signal as a fixed concentration of **Fl-NB-72** was displaced by increasing concentrations of compound. Representative examples are shown in Fig. 2b. The K_i_ values, calculated from the IC_50_ values, are given for all compounds in Supplementary Table 1. The mono and diamines and their methiodide salts have high binding affinity for FOXM1, with K_i_ values in the submicromolar range; the methiodide salts of the diamines bind with higher affinity than the diamines themselves.

We also examined the effect of these compounds on the level of intracellular FOXM1 and on the proteolytic lability of FOXM1 in cell extracts. Treatment of cell extracts with **NB-73** rendered the FOXM1 protein more readily proteolyzed by pronase (Fig. 2c), suggesting that binding of compound to FOXM1 destabilizes its structure and increases the ease with which it can be degraded by pronase. Likewise, exposure of cells (MDA-MB-231 or MCF7) to **NB-55, NB-73, or NB-115** reduced the intracellular level of FOXM1 over time (Fig. 2d, e), whereas the intracellular level of a different forkhead protein, FOXA1, was not altered by these compounds (Fig. 2e). Of interest, intracellular FOXM1 protein level was minimally affected by **FDI-6** exposure of cells (Fig. 2d), suggesting that our compounds and **FDI-6** have somewhat different mechanisms of action. Also, as shown in Fig. 2f, cotreatment of cells with the proteasome inhibitor MG132 reversed the downregulation of cellular FOXM1 by **NB-73**.

### RNA-Seq analysis of the effects of compounds and of siFOXM1 on global FOXM1 gene regulation

**Fig. 3:**
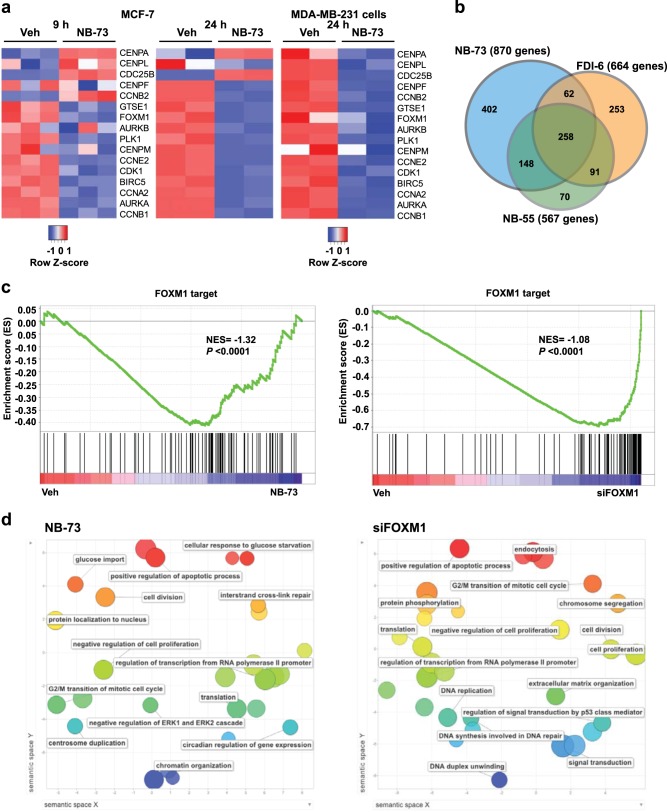
RNA-Seq analysis of the effects of compounds on gene expression in breast cancer cells. **a** Regulation of FOXM1-signature genes by **NB-73** (4 µM) in MCF7 cells at 9 h and 24 h, and by **NB-73** treatment (1.5 µM, 24 h) in MDA-MB-231 cells. **b** Venn diagram showing overlap of genes with *p* < 0.05 and regulated more than 2-fold by **NB-73**, **NB-55** and **FDI-6** (20 µM) in MCF7 cells, 9 h treatment. **c** Gene Set Enrichment Analysis (GSEA) showing Enrichment Scores. MDA-MB-231 cells treated with **NB-73** (1.5 µM, 24 h) or siFOXM1 (25 nM, 72 h). Gene sets used FOXM1 genes derived from four independent datasets of FOXM1 cistromes.^19,52^ NES, normalized enrichment score. **d** REVIGO (Reduce and Visualize Gene Ontologies) analyses showing biological process gene ontologies most impacted by MDA-MB-231 cell treatment with **NB-73** or siFOXM1. Circle sizes represent GO term gene count; colors represent similarity along Semantic Space Y.

We next used RNA-Seq to examine the effects of these compounds on gene regulation more globally. Because full FOXM1 depletion results in mitotic catastrophe and cell death,^20^ we used concentrations of compounds that suppressed cell proliferation by about 60%. As seen in the heat maps in Fig. 3a, **NB-73** regulated FOXM1 RNA-signature genes at 9 h and even more strongly at 24 h in MCF7 and MDA-MB-231 cells. Further, as shown in the Venn diagram (Fig. 3b), there was extensive overlap in the genes regulated more than 2-fold and with FDRs < 0.05 by **NB-73**, **NB-55**, and **FDI-6**. Notably, 72% of genes regulated by **NB-55** and 48% of genes regulated by **FDI-6** overlapped with **NB-73** regulated genes, indicating that these compounds regulated many similar genes. Gene Set Enrichment Analyses (GSEA) and Enrichment Scores for gene regulations by **NB-73** or siFOXM1 treatment of cells are shown in Fig. 3c. These analyses testing the differential gene expression data against gene sets consisting of FOXM1 target genes revealed negative enrichment scores, indicating that **NB-73** and siFOXM1 downregulate expression of genes in the FOXM1 cistrome. The major categories of gene regulations, identified by Gene Ontology analysis and shown in the reduce and visualize gene ontologies (REVIGO) plots (Fig. 3d and Supplementary Fig. 2), included proliferation, G2/M transition of mitotic cell cycle, apoptosis, regulation of transcription, DNA replication and DNA repair, activities well known to be under FOXM1 regulation.^21^

### Effects of compounds on the cell cycle, apoptosis, and on nuclear localization of FOXM1

**Fig. 4:**
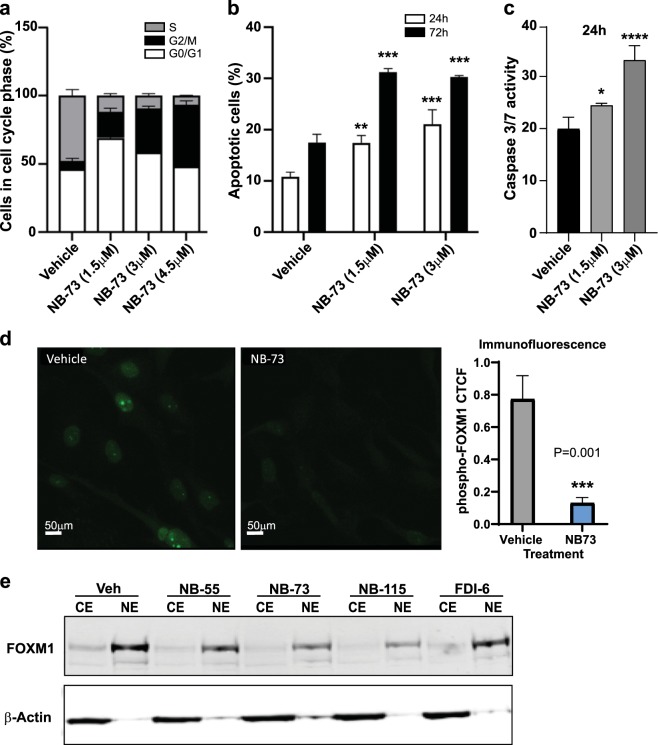
Compound **NB-73** increases the percent of cells in G2/M and reduces the percent of cells in S phase of the cell cycle, and increases apoptosis, but does not change the nuclear localization of the FOXM1 present. **a** MDA-MB-231 cells were synchronized by double thymidine block and were released for 24 h with or without treatment with vehicle or **NB-73** (at 1.5, 3.0, or 4.5 µM). The percent of cells in different phases of the cell cycle were monitored by flow cytometry. *n* = 4 experiments, mean + SD. **b** Cells were treated with or without **NB-73** (at 1.5 or 3 µM) for 24 h or 72 h and the percent of apoptotic cells was monitored by AnnexinV/propidium iodide staining and flow cytometry. *n* = 3 experiments, mean + SD. **c** Caspase 3/7 activity was monitored after cell treatment with or without **NB-73** (at 1.5 or 3 µM) for 24 h using Caspase Glo assay. *n* = 2 experiments, mean + SD; *t*-test **p < 0.01; ***p < 0.001. **d NB-73** treatment (2 µM for 24 h) of MDA-MB-231 cells results in a decrease of p-FOXM1, observed by immunofluorescent staining (Scale bar = 50 µm). Nuclei from four fields for vehicle and **NB-73** treated cells were analyzed using the Image J fluorescent analysis tool, the average CTCF (corrected total cell fluorescence) was calculated, and data were statistically analyzed by paired *t*-test. **e** Treatment with **NB-55, NB-73**, or **NB-115** or **FDI6** for 24 h does not change the intracellular localization of FOXM1, which remains largely nuclear, as observed by Western blot of nuclear extracts (NE) and cytoplasmic extracts (CE) of cells after 24 h. By contrast, β-Actin is largely in the CE, as expected.

As seen in Fig. 4, the compounds had a marked effect on the cell cycle and also on the proportion of cells undergoing apoptosis. In Fig. 4a, cells were synchronized by double thymidine block and were then released for 24 h with or without treatment with **NB-73** at three different concentrations (1.5, 3.0, and 4.5 µM). Flow cytometry analysis (Fig. 4a and Supplementary Figs 3 and 4) showed that compound treatment resulted in a large increase in the percent of cells in G2/M, and decrease in the percent of cells in S phase, consistent with the compounds inhibiting FOXM1 activity.^9,22^ FOXM1 is a key regulator of G1/S and G2/M transitions and M phase progression and FOXM1 binds to many G2/M promoters.^8,9^ Treatment with **NB-73** also increased the percent of apoptotic cells observed at 24 h or 72 h (Fig. 4b) and caspase 3/7 activity (Fig. 4c).

Of note, treatment with **NB-73** decreased the expression of phospho-FOXM1, considered the biologically active form of FOXM1, as observed by immunofluorescence (Fig. 4d), consistent with western blot findings in Fig. 2d, e that **NB-73** treatment decreased FOXM1. Despite the change in amount of FOXM1 protein with **NB-73** exposure, FOXM1 protein was always found in the nucleus and showed the same, greater than 90%, nuclear localization as observed in control vehicle treated cells both by immunofluorescence (Fig. 4d) and by biochemical fractionation of cells into nuclear and cytoplasmic fractions (Fig. 4e).

### Pharmacokinetics of compounds

**Fig. 5:**
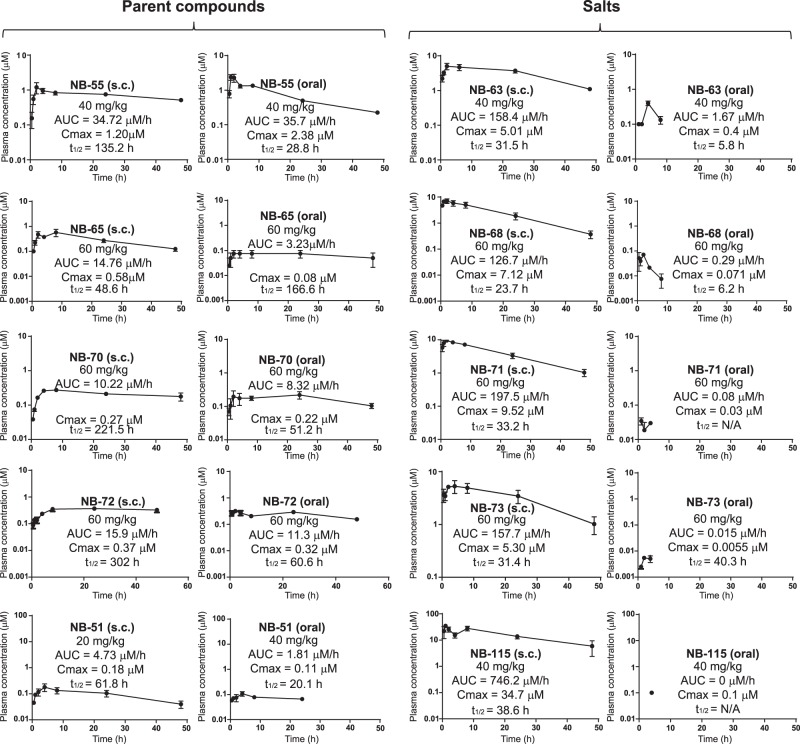
Pharmacokinetics and half-lives of compounds in mice after s.c. or oral administration of amine compounds and their respective methiodide salts. Pairs are **NB-55** and **NB-63**; **NB-65** and **NB-68**; **NB-70** and **NB-71**; **NB-72** and **NB-73**; and **NB-51** and **NB-115**. PK was studied after single dose administration via s.c. injection or oral gavage at the doses indicated. Multiple plasma samples were collected from each mouse (*n* = 4) over the course of 48 h after compound was administered. Compounds were quantified using LC-MS/MS. The data were fitted to a non-compartment PK model (error bars are ± SD).

In pharmacokinetic (PK) studies in mice, we found that some compounds showed very good subcutaneous and oral bioavailability (Fig. 5). Examination of compound half-lives and accumulation in plasma after a single subcutaneous injection or oral administration of the parent mono- and diamines (**NB-55**, **65**, **70**, **72**, and **51**) revealed good and rather equivalent PK by either route of administration. It is striking that the PK properties of the monoamine **NB-55**, particularly after oral dosing, were markedly superior to those of the diamines. Of note as well, conversion of the mono- and diamines to their methiodide salts improved their PK behavior after subcutaneous administration; the oral bioavailability of these salts, however, was diminished (Fig. 5). Hence, after s.c. injection of **NB-63**, **NB-68**, **NB-71**, **NB-73**, and **NB-115**, very high blood levels of compound were observed, followed by long half-lives (from ~25–40 h, Fig. 5), whereas only very low levels were found after oral administration. In contrast, the FOXM1 inhibitor **FDI-6** achieved only very low blood levels after s.c. administration at 20 mg/kg, and was undetectable after oral administration at 40 mg/kg (and therefore not shown) (Supplementary Fig. 4).

### Efficacy of compounds in suppressing the growth of human breast tumor xenografts and FOXM1-regulated gene expression in tumors

**Fig. 6:**
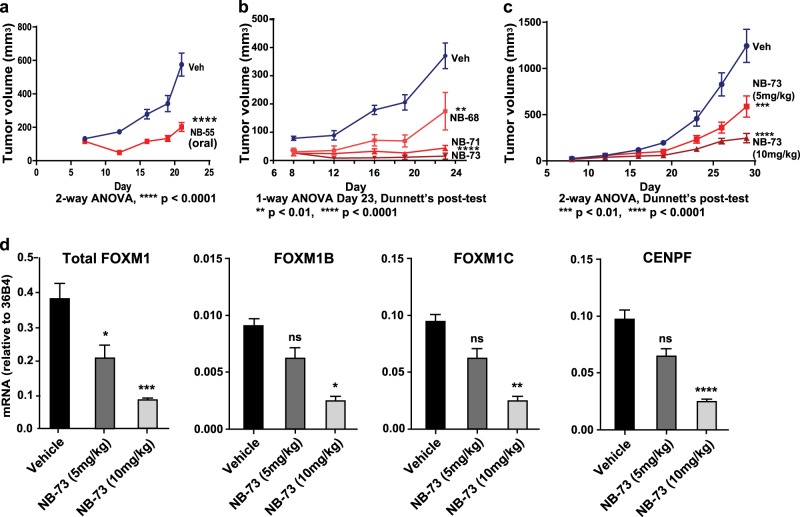
Compounds **NB-55, NB-68**, **NB-71**, and **NB-73** suppress breast tumor xenograft growth and the expression of FOXM1-regulated genes. Human breast cancer DT22 cells were injected into the mammary fat pad of intact 7-week-old NSG mice and mice bearing DT22 tumors were dosed in **a** daily and then every other day from day 7 on with 100 mg/kg of **NB-55** or control vehicle by oral gavage; or **b** with **NB-68**, **NB-71**, or **NB-73** by s.c. injection at 40 mg/kg daily for 4 days and then 20 mg/kg every third day until day 13 and then 10 mg/kg every third day, or with control vehicle. Tumor volumes in Veh and compound treated animals were monitored (2-way ANOVA, Dunnett’s post-test, **P < 0.01). In **c**, animals were treated with low doses of **NB-73** (5 or 10 mg/kg s.c. daily and then every other day after day 21). Volumes of tumors in Veh and compound treated animals were monitored (2-way ANOVA, Bonferroni post-test, ****P < 0.0001, *n* = 8 per group, error bars are ± SD). **d** At the end of treatments, tumors from panel C were processed for gene expression analysis by q-PCR. (*t*-test, *p < 0.05; **p < 0.01; ***p < 0.001; ****P < 0.0001, *n* = 8 per group, error bars are ± SD).

Studies in female NOD/SCID-gamma (NSG) mice showed that the monoamine **NB-55**, when given orally daily, very effectively suppressed the growth of human breast tumor xenografts (Fig. 6a). We also tested lower doses of the diammonium salts **NB-68**, **NB-71**, and **NB-73** as tumor suppressive agents, since these compounds had higher inherent cellular potencies and achieved higher blood levels after s.c. dosing than did **NB-55**. At 20 mg/kg every other day for 10 days and then 10 mg/kg every other day subsequently, **NB-68**, **NB-71**, and **NB-73** were found to greatly suppress tumor growth (Fig. 6b). Because of their effectiveness, we next treated animals with 5 and 10 mg/kg of **NB-73** s.c. daily and then every other day starting on day 21. As seen in Fig. 6c, **NB-73** reduced tumor growth at 5 mg/kg and more markedly suppressed growth at 10 mg/kg. Accompanying this suppression of tumor growth by **NB-73**, the expression of FOXM1-regulated genes, including the FOXM1 gene itself, was reduced in tumors in a dose-dependent manner, with all gene expressions being greatly reduced upon treatment with 10 mg/kg **NB-73** (Fig. 6d). Low doses of **NB-63** and **NB-115**, compounds that showed good PK properties with high blood levels and long half-lives, were also effective in suppressing the growth of tumor xenografts (Supplementary Fig. 5) and had little impact on animal body weight.

## Discussion

This study presents a new class of 1,1-diarylethylene mono-and diamine compounds that function as effective suppressors of FOXM1 activities. These compounds and their methiodide salts bind directly to FOXM1 protein, labilize it towards proteolytic degradation, and reverse patterns of FOXM1-regulated gene expression. They also suppress breast cancer cell proliferation and increase apoptosis, and they retard the growth of breast xenograft tumors in an experimental preclinical mouse model where we show that FOXM1-mediated gene expression is suppressed in these growth-inhibited tumors.

Several lines of evidence indicate that our compounds are targeting FOXM1 and fulfill characteristics expected for validated chemical probes for this target protein.^23,24^ By a cell-free FRET binding assay, we show that the FOXM1 inhibitory compounds bind directly to FOXM1 with affinities commensurate with their cellular potencies. Treatment of cells with these compounds reduced the intracellular level of FOXM1 and exposure of cell extracts to these compounds increased FOXM1 degradation by pronase, as observed in the drug affinity responsive target stability (DARTS) protease sensitivity assay.^25^ This implies that compound binding is perturbing the structure of FOXM1 to make it more readily proteolyzed, which matches the fact that they also decrease the intracellular level of FOXM1 protein. Of interest, antiestrogen SERD compounds also inhibit and promote degradation of their target protein, ERα^26,27^ by conformational disordering of the ligand-binding domain. Similar order-to-disorder conformational changes in FOXM1, recently shown to regulate its transcriptional activity, are likely being affected by our compounds in ways that also block FOXM1 activity and accelerate its degradation.^28^ Also, gene regulation by **NB-73** and **NB-55**, studied in greatest detail, showed substantial overlap with that of **FDI-6**, shown previously to target FOXM1,^16^ and with siFOXM1 treatment of cells. Cell cycle analysis, showing an increase in the proportion of cells in G2/M after treatment with NB-73, is also consistent with FOXM1 activity being inhibited by NB-73. However, we do not yet know if our compounds affect only FOXM1, although they show selectivity in reducing intracellular FOXM1 without changing the intracellular level of another important forkhead transcription factor, FOXA1. In addition to regulating the expression of many genes that promote tumorigenesis and cancer progression, FOXM1 can also influence these processes through its interactions with other key cellular proteins such as SMAD3 and β-catenin.^3^ Possible effects of our NB compounds on altering the activity of FOXM1 through these protein interactions remain to be studied in future investigations.

Of the amine compounds studied that showed good potency (IC_50_ values less than 0.5 μM) and efficacy in cells in culture, several had good in vivo PK properties. Although **NB-55** and **NB-65** showed good PK properties (half-lives and blood levels achieved) after s.c. administration, only **NB-55** showed good PK properties after oral administration. Notably, the salts of all of the amine compounds had greatly (5–50×) elevated blood levels and long half-lives (t_½_ = 24–39 h) by s.c. route. However, these salts showed very low bioavailability (blood levels) by oral route. Because of their high and prolonged blood levels, **NB-63**, **NB-68**, **NB-71**, **NB-73**, and **NB-115** were able to greatly suppress tumor growth in vivo using low doses (2–20 mg/kg) administered s.c. daily or every other day. By contrast, **NB-55** required daily s.c. or oral treatment at 100 mg/kg for effective tumor suppression, whereas 20 or 40 mg/kg doses were found to be only marginally effective (data not shown), consistent with its good but not outstanding blood levels and its 5-fold reduced potency in suppression of cell proliferation, as seen in dose-response studies in cells in culture. Of note, we observed that **FDI-6**, for which no prior in vivo data have been published,^16^ showed potency similar to **NB-55** in cells in culture but achieved only very low blood levels after s.c. or oral administration.

Of interest, the FOXM1 activity-regulating compounds described in this manuscript were able to function as suppressors of breast cancer cell proliferation and the expression of FOXM1-signature genes and gene ontologies in a broad range of breast cancer subtypes, both hormone receptor-positive (such as MCF7 and tamoxifen-resistant MCF7) and triple negative (such as DT22 and MDA-MB-231), as well as in BT474 (ER-positive, HER2-positive) cells, where similar IC_50_ concentrations for inhibition of cell proliferation and FOXM1-regulated gene expressions were observed. Indeed, there is considerable evidence for the deleterious impact of high tumor FOXM1 on patient clinical outcome in ER-positive breast cancers,^15,29,30^ in HER2-positive breast cancers,^31^ and in triple negative breast cancers.^32^ FOXM1 is also a key player in many other cancers, including glioblastoma, ovarian, gastrointestinal, non-small cell lung cancers, and pancreatic ductal adenocarcinoma,^6,7,11,14,33,34^ for which there are currently few optimal treatments. Hence, compounds that inhibit FOXM1 activities might prove to be broadly useful in a variety of cancer types.

In this study, we found that these compounds were effective antitumor agents when administered alone at low micromolar doses in several breast cancer subtypes; nevertheless, it will be of interest to test them in combination treatments targeting ER-positive, recurrent metastatic endocrine therapy-resistant breast cancers and aggressive triple negative breast cancers, when administered with other current standard-of-care treatments. For example, combinations of these FOXM1 inhibitors with Fulvestrant, Letrozole, PI3K inhibitors, or CDK4/6 inhibitors^35,36^ might enable improved tumor sensitivity and response, since we and others have shown that knockdown of FOXM1 or inhibition of FOXM1 activity with an ARF inhibitory peptide in cells in culture could restore sensitivity to tamoxifen in tamoxifen-resistant breast cancer cells.^15^ Since FOXM1 has been found to reduce cancer responsiveness to chemotherapeutic agents and to radiation,^13,37^ inhibition of FOXM1 activity in triple negative breast cancers might likewise reduce the amounts of chemotherapy drugs or radiation needed, thereby improving cancer treatment and perhaps reducing undesirable side effects of many current drug therapies.

The compounds we have developed suppress FOXM1 activities and have favorable pharmacokinetic properties. Hence, these new compounds offer intriguing translational opportunities for the development of new anticancer agents, either as mono or combination therapies targeting FOXM1 actions that drive aggressive forms of breast cancer, as well as other cancers.

## Methods

### Cell lines and cell culture methods

All breast cancer cell lines were obtained from the ATCC and were maintained and cultured as described.^15,38,39^ DT22 cells were derived from a human triple negative invasive ductal breast carcinoma and were grown in culture as described.^40^ All cells were tested for mycoplasma using Real-Time PCR Mycoplasma Detection Kit (Akron Biotech, Boca Raton, FL).

### Chemical synthesis of compounds

Full details on the preparation and spectroscopic characterization of all compounds are given in the Supplementary Information.

### Cell viability assay

WST-1 assay (Roche, Basel, Switzerland) was used to quantify cell viability as described.^38^ Absorbance was measured at 450 nm using a VICTOR X5 PerkinElmer 2030 Multilabel Plate Reader. All assays were performed in triplicate and analyzed using Graph Pad Prism 8.0.

### Western blot and Immunofluorescence assays

For Western blot analysis, whole-cell extracts were prepared using 1X RIPA lysis buffer (Thermo Fisher) supplemented with 1X protease inhibitor cocktail (Millipore Sigma). Proteins were separated on 4–12% SDS-PAGE gels and transferred to nitrocellulose membranes. In some cases, treatments with compound were done in the presence of the proteasome inhibitor MG132 (Calbiochem) as described in the figure legend. Western blotting used antibodies against FOXM1 (Abcam 184637, 1:1000; Cell Signaling Technologies Catalog #5436, D12D5, 1:1000), ERα (Santa Cruz Catalog #sc543, HC-20, 1:1000), FOXA1 (Abcam 23738, 1:1000), and β-actin (Millipore-Sigma A2228, 1:10,000) as an internal loading control. Both IRDye 800 CW goat anti-rabbit secondary antibody (LI-COR, Cat# 926-32211) and IRDye 680 CW goat anti-mouse secondary antibody (LI-COR, Cat# 926-68070) were diluted (1:5000) for incubation with the blots. Band intensities were analyzed with Licor Odyssey 2.1 software. All blots shown together derive from the same experiment and were processed in parallel. Full uncropped images of blots are shown as supplementary figures in the Supplementary Information file. Molecular weight markers were Rainbow markers from GE Healthcare (38–225 kDa) or Precision Plus Dual Color Markers from Biorad (37–250 kDa).

Immunofluorescent staining and confocal microscopy were performed as described.^41^ Briefly, for immunofluorescence detection of FOXM1 in cells, cells were grown in 8-well chamber slides (Ibidi, Verona, WI), fixed in 4% formaldehyde, and permeabilized with 0.1% Triton-X100. The cells were then blocked, treated with primary antibody to phospho-FOXM1 (Cell Signaling Technology Catalog #14655, D9M6G 1:100) followed by a fluorescently-tagged secondary antibody (Jackson Immunoresearch 711-546-152, 1:500), and nuclei were counterstained with DAPI before imaging. Three dimensional Z stacks were compressed, the compressed images were exported to Image J, and the corrected total cell fluorescence (CTCF) was calculated for each individual cell or nucleus. Data were statistically analyzed as indicated in the figure legend.

### Fluorescence binding assays with FOXM1

Competitive binding assays of compound binding to FOXM1 by tr-FRET were done in FRET buffer (20 mM Tris, pH 7.5, 50 mM NaCl, 0.01% NP-40 detergent, 10% glycerol) with 0.3 mg/ml ovalbumin and 0.1 mM butylated hydroxy-anisole added fresh daily. Solutions of the protein, fluorescent probe and compound dilutions were prepared at 3× concentrations, the final dilution taking place as they were mixed together in equal portions on the microtiter plate.

A stock solution of biotin-FOXM1 was prepared and stored at 5.6 μM at −80 °C. For the assay, it was diluted to be 5 nM final concentration, with 1.25 nM tetravalent streptavidin- terbium (SaTb). The compounds were prepared at 7 × 10^−4^ M in DMF (dimethylformamide) and then serially diluted into FRET buffer plus 2% DMF to ensure solubility. Final concentrations were 10^−5^ M to 10^−9^ M, with the last point being buffer only, no compound. Fluorescein-**NB-72** (**Fl-NB- 72**) was prepared at 1 mM in DMF and stored at −20 °C. It was diluted into FRET buffer to give 100 nM in the assay. Incubations were done in duplicate on black Molecular Devices 96-well microtiter plates. 5 μl of **Fl-NB-72** and 5 μl of the compound dilutions, were mixed together on the plate. To this was added 5 µl biotin-FoxM1 and incubated in the dark at room temperature, for 1 h. Time-resolved Förster resonance energy transfer (tr-FRET) measurements were with a Victor X5 plate reader (Perkin Elmer), with an excitation filter at 340/10 nm and emission filters for terbium and fluorescein at 495/20 and 520/25 nm, respectively, with a 100-μs delay in data acquisition. To correct for background signals, biotin-FOXM1 was omitted, and diffusion-enhanced FRET incubations were done in duplicate on black Molecular Devices 96-well microtiter plates. Five microliter of **Fl-NB-72** and 5 μl of the compound solutions were mixed together and then 5 µl of FRET buffer plus SaTb was added, mixed and incubated in the dark at room temperature, for 1 h. The tr-FRET signals measured as above were subtracted as a background from the first incubations that contained biotin-FOXM1. Graphs were prepared using Graph Pad/Prism 4.

The Ki was calculated by the Cheng- Prusoff equation (Ki (compound) = IC50 (compound)/(1 + T0/Kd (Fl-NB-72)); T0 is the concentration of **Fl-NB-72** and Kd(**Fl-NB-72)** is its binding affinity for FOXM1, determined below.

In direct binding experiments for determination of the Kd for **Fl-NB-72**, the **Fl-NB-72** ligand was diluted into FRET buffer + 2% DMF, to give concentrations 3× of final. The biotin-FOXM1 was diluted to a concentration of 3 × (5 nM + 1.25 nM SaTb). 5 μl of each of these were mixed on a black Molecular Devices microtiter plate with 5 μl of FRET buffer, so that each component was diluted 3×. Each point was prepared in duplicate, mixed, and incubated for 1 h at room temperature in the dark. tr-FRET was measured on a Victor X5 microtiter plate reader with settings as given above.

### Drug affinity responsive target stability (DARTS) assay

The DARTS assay was performed as described^25^ to examine the effect of compounds on the stability of FOXM1 to proteolysis by exogenous pronase. Cell lysates were incubated without or with compound for 1 h at room temperature, and with varying concentrations of pronase (none; 1:10^3^; 1:3 × 10^3^; 1:5 × 10^3^; 1:10^4^; and 1:10^5^ dilution of a 12.5 mg/ml pronase solution) for an additional 30 min at room temperature. Proteins were then separated on 4–12% SDS-PAGE gels and gels were exposed to FOXM1 antibody (Genetex GTX102170, 1:750) and β-actin antibody (1:500, mouse monoclonal).

### Cell cycle analysis

Cells were synchronized by double thymidine block prior to cell cycle analysis by Propidium Iodide (PI) staining. Briefly, the cells were blocked with 2 mM thymidine for 18 h, then released with fresh media for 8 h followed by re-blocking with thymidine for another 16 h. Following the second block, the cells were released with or without treatments with the NB compounds and cells were collected and fixed in 70% ethanol at the time points indicated. After alcohol fixation for 2 h, the cells were washed with cold 1XPBS followed by staining with 50 µg/ml PI solution in PBS with addition of 5 µg/ml RNAse A for at least 4 h. The cells were then analyzed by the Flow Cytometry analyzer BD LSR II for percentage of cells in G1/G0, S, and G2/M phases of the cell cycle.

### Apoptosis analysis

The cells were analyzed for percentage of apoptotic cells following 24 h or 72 h of vehicle or compound treatment by staining with the Alexa Fluor® 488 Annexin V/Dead Cell Apoptosis Kit (Thermo Fisher) and flow cytometry according to the manufacturer’s protocol. Caspase activity in the cells after treatment with control vehicle or compounds was determined using the Caspase-Glo 3/7 Assay system (Promega) in a 96-well format following the manufacturer’s instructions. Supplementary Figs 6 and 7 exemplify the gating strategy used for the cell cycle and apoptosis flow cytometry analyses.

### Cytoplasmic and Nuclear extract preparation

Cells treated with vehicle or compounds were collected for preparation of cytoplasmic and nuclear fractions. Briefly, cells were washed with ice cold PBS and centrifuged at 6000 rpm for 5 min. Following removal of the supernatant, the cell pellets were resuspended in 150–200 µL of cold CE buffer (HEPES [10 mM] pH 7.9, KCI [10 mM], EDTA [0.1 mM], NP-40 0.3% (added just before use) and 1X protease inhibitors (added just before use)) and incubated on ice for 5 min. Cells were then centrifuged at 3000 rpm for 5 min and the supernatant (cytoplasmic extract) was harvested. The pellets were washed twice in 100 µl of CE buffer without NP-40. After removal of supernatant, pellets were resuspended in 20–40 µl of NE buffer (HEPES [20 mM] pH 7.9, NaCl [0.4 mM], EDTA [1 mM], glycerol 25%, 1X protease inhibitors (added just before use)) and incubated on ice for 10 min, followed by centrifugation at 14,000 rpm for 5 min and harvest of supernatant (nuclear extract). All centrifugations were done at 4 C.

### RNA isolation and real-time PCR

Total RNA was isolated using TRIzol (Invitrogen) and reverse transcribed using MMTV reverse transcriptase (New England BioLabs). Real-time PCR was performed using SYBRgreen PCR Master Mix (Quantabio) as described.^15^ Relative mRNA levels of genes were normalized to the housekeeping gene 36B4, and fold-change calculated relative to the vehicle treated samples. Results are the average ± SD from at least two independent experiments carried out in triplicate. Primer sequences for the genes studied were obtained from the Harvard Primer Bank. Sequences are available on their website.

### RNA-Seq transcriptional profiling and gene ontology analysis

For gene expression analysis, total RNA was extracted from cells using Trizol reagent and further cleaned using the Turbo DNase and RNAqueous kits (ThermoFisher). Cells were treated with Veh (0.1% EtOH), or with the compounds indicated for 9 h or 24 h. Once the sample quality and replicate reproducibility were verified, samples from each group were subjected to sequencing. RNA at a concentration of 100 ng/µl in nuclease-free water was used for library construction. cDNA libraries were prepared with the mRNA-TruSeq Kit (Illumina, Inc.). In brief, the poly-A containing mRNA was purified from total RNA, the RNA was fragmented, double-stranded cDNA was generated from fragmented RNA, and adapters were ligated to the ends.

The paired-end read data from the HiSeq 4000 were processed and analyzed through a series of steps. Base calling and de-multiplexing of samples within each lane were done with Casava 1.8.2. The RNA sequences were prepared with Illumina’s TruSeq Stranded mRNAseq Sample Prep kit. Reads were trimmed of adapters and low expression data using Trimmomatic version 0.38.^42^ The STAR alignment tool version 2.5.3a was used to align the sequenced reads to the GRCh37 human genome from Ensembl.^43^ Gene counts were calculated using subread version 1.5.2.^44,45^ The edgeR Bioconductor package in R was used for normalization and differential expression analysis. Default normalization methods were used, specifically trimmed mean of M values or TMM was used to calculate the normalized expression values. This method calculates the weighted trimmed mean of the log expression ratios in a gene-wise fashion.^46,47^ We considered genes with fold-change > 2 and *p*-value < 0.05 as statistically significant, differentially expressed.

Gene Set Enrichment Analysis (GSEA)^48^ was used for examination of our genome-wide expression profiles. Overrepresented gene ontology (GO) biological processes were determined by the web-based DAVID Bioinformatics Resources database.^49,50^ REVIGO (Reduce and Visualize Gene Ontologies) was utilized to visualize overrepresented GO biological processes.^51^

### Pharmacokinetic studies

All experiments involving animals were conducted in accordance with National Institutes of Health (NIH) standards for the care and use of animals, with protocols approved by the University of Illinois IACUC. The pharmacokinetics of compounds were monitored after single dose administration into female CD1 mice (7–9 weeks of age) via s.c. injection or oral gavage, as described.^39^ For s.c. injection, each compound was dissolved in DMSO and then mixed with corn oil for a total injection volume of 100 μL (10% DSMO + 90% corn oil) per mouse. For oral gavage, compounds were administered in a 200 µL formulation of 9/0.5/0.5/90 parts of PEG400/Tween80/Povidone/0.5% Carboxymethylcellulose in DI water. Multiple plasma samples were collected from each mouse (*n* = 4 for each experiment) over the course of 48 h after compound administration. Compounds were quantified by LC-MS/MS at the University of Illinois Metabolomics Core Facility. The data were fitted to a non-compartment model.

### In vivo breast cancer xenograft studies

For examination of the effects of compounds on breast tumor growth suppression, intact female NOD/SCID-gamma (NSG) mice were used as detailed previously.^39^ DT-22 breast cancer cells (1 × 10^6^ cells/mouse) were injected s.c. into the right axial mammary gland. Mice received s.c. injection or oral gavage daily or every second or third day as indicated, with vehicle or treatment compound, and tumor volume (length × width^2^/2) was monitored over time.

## Supplementary information

Supplementary Information

## Data Availability

Datasets supporting Figs 1–6, Supplementary Table 1 and Supplementary Figs 1, 2, 4 and 5 in this published article are publicly available in the figshare repository: 10.6084/m9.figshare.10052219.^17^ RNA-Seq data of the effects of compounds and of siFOXM1 on global *FOXM1* gene regulation, are publicly available in the NCBI Gene Expression Omnibus (GEO) repository: https://identifiers.org/geo:GSE132343.^21^ Uncropped Western blots are available as part of the supplementary information (Supplementary Fig. 8).
