# Supplementary material for: Suppression of FOXM1 activities and breast cancer growth in vitro and in vivo by a new class of compounds
Source: NPJ Breast Cancer. 2019 Nov 29;5:45. doi: 10.1038/s41523-019-0141-7 (PMC6884575; doi:10.1038/s41523-019-0141-7)
Supplement: Supplementary file 1 — Supplementary Information [file 41523_2019_141_MOESM1_ESM.pdf]

Supplementary Information for

Suppression of FOXM1 activities and breast cancer growth *in vitro* and *in vivo* by a new class of compounds

Yvonne Ziegler[1], Mary J. Laws[1], Valeria Sanabria Guillen[1], Sung Hoon Kim[2], Parama Dey[1], Brandi P. Smith[3], Ping Gong[1], Noah Bindman[2], Yuechao Zhao[1], Kathryn Carlson[2], Mayuri A. Yasuda[1], Divya Singh[1], Zhong Li[4], Dorraya El-Ashry[5], Zeynep Madak-Erdogan[3], John A. Katzenellenbogen[2], and Benita S. Katzenellenbogen*[1]

Supplementary Information

Table and Figures and Experimental Methods

**Supplementary Table 1. Affinity of compounds and FDI-6 for binding to FOXM1 protein**		
Compound	*[Cpd No.] Ki (μM)[a]*	
	Amine	*Methiodide Salt*
Monoamine		
Cl & 5-ring	[**NB-55**] 0.23	[**NB-63**] 0.076
Diamines		
5-ring	[**NB-65**] 0.19	[**NB-68**] 0.051
6-ring	[**NB-70**] 0.10	[**NB-71**] 0.011
7-ring	[**NB-72**] 0.065	[**NB-73**] 0.014
Et_2 amine	[**NB-51**] 0.13	[**NB-115**] 0.040
**FDI-6**	0.143	

[a]K_i values are calculated from IC_{50} values, as described in the Methods.

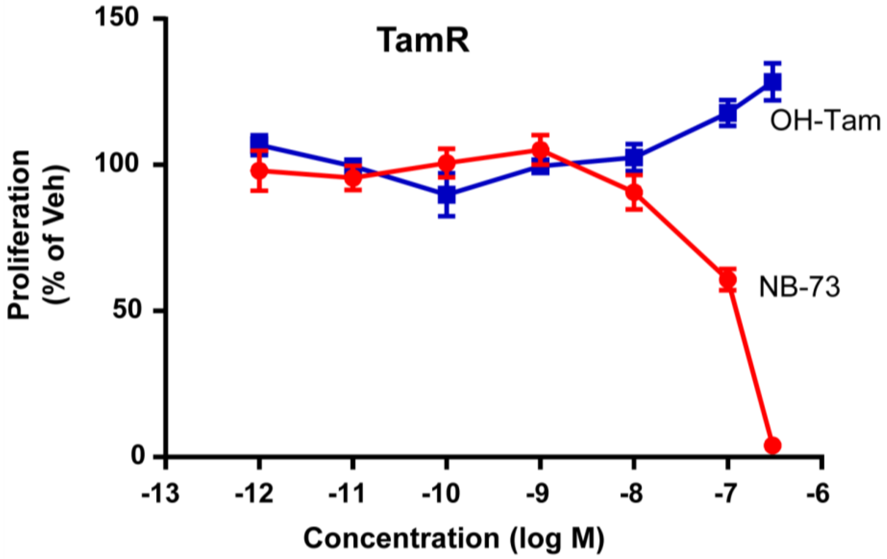

Supplementary Figure 1. Compound **NB-73** inhibits the proliferation of tamoxifen-resistant MCF7 cells. Cells were treated with **NB-73** or trans-hydroxytamoxifen (OH-Tam) for 6 days and cell numbers were monitored. Values are mean ± SD of three separate assays.

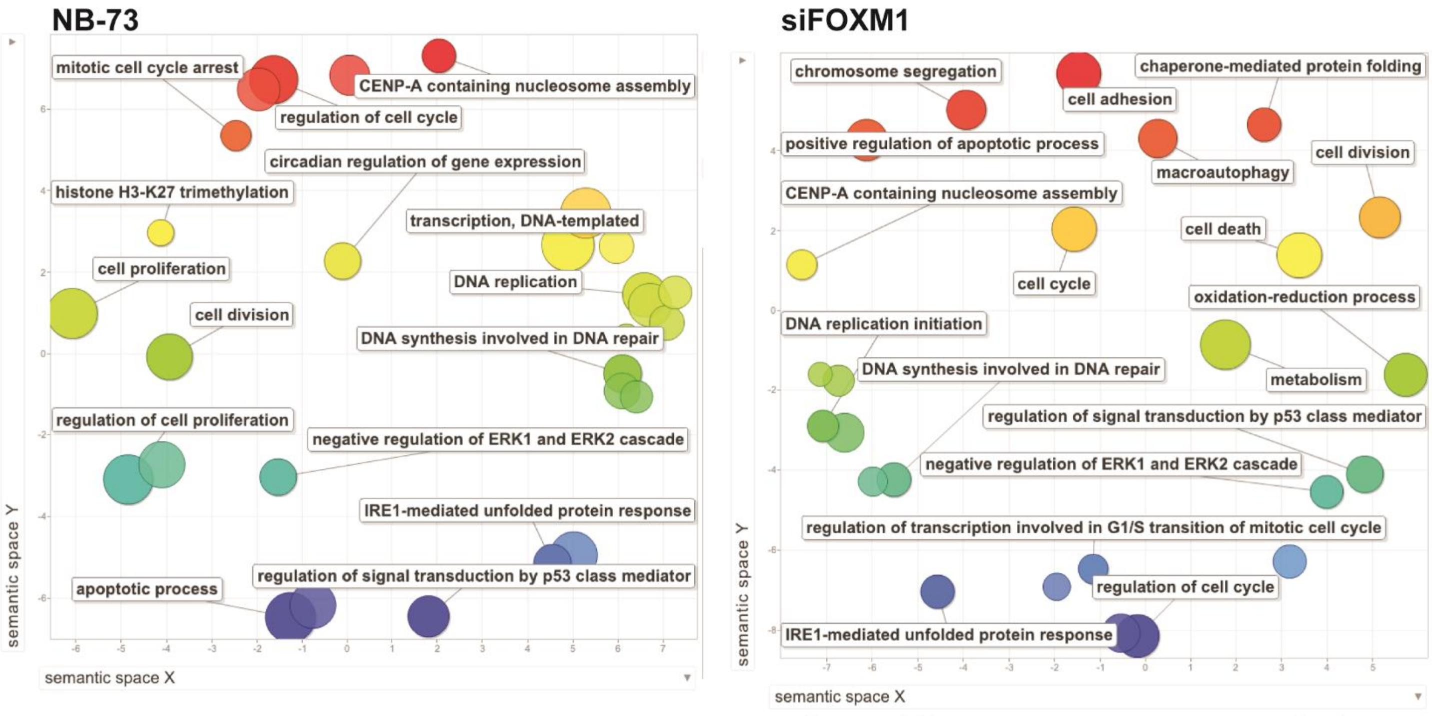

Supplementary Figure 2. REVIGO (Reduce and Visualize Gene Ontologies) display of gene ontologies most affected by MCF7 cell treatment with **NB-73** (4 μM for 24 h) or siFOXM1 (25 nM for 72 h). Circle sizes represent GO term gene count; colors represent similarity along Semantic Space Y.

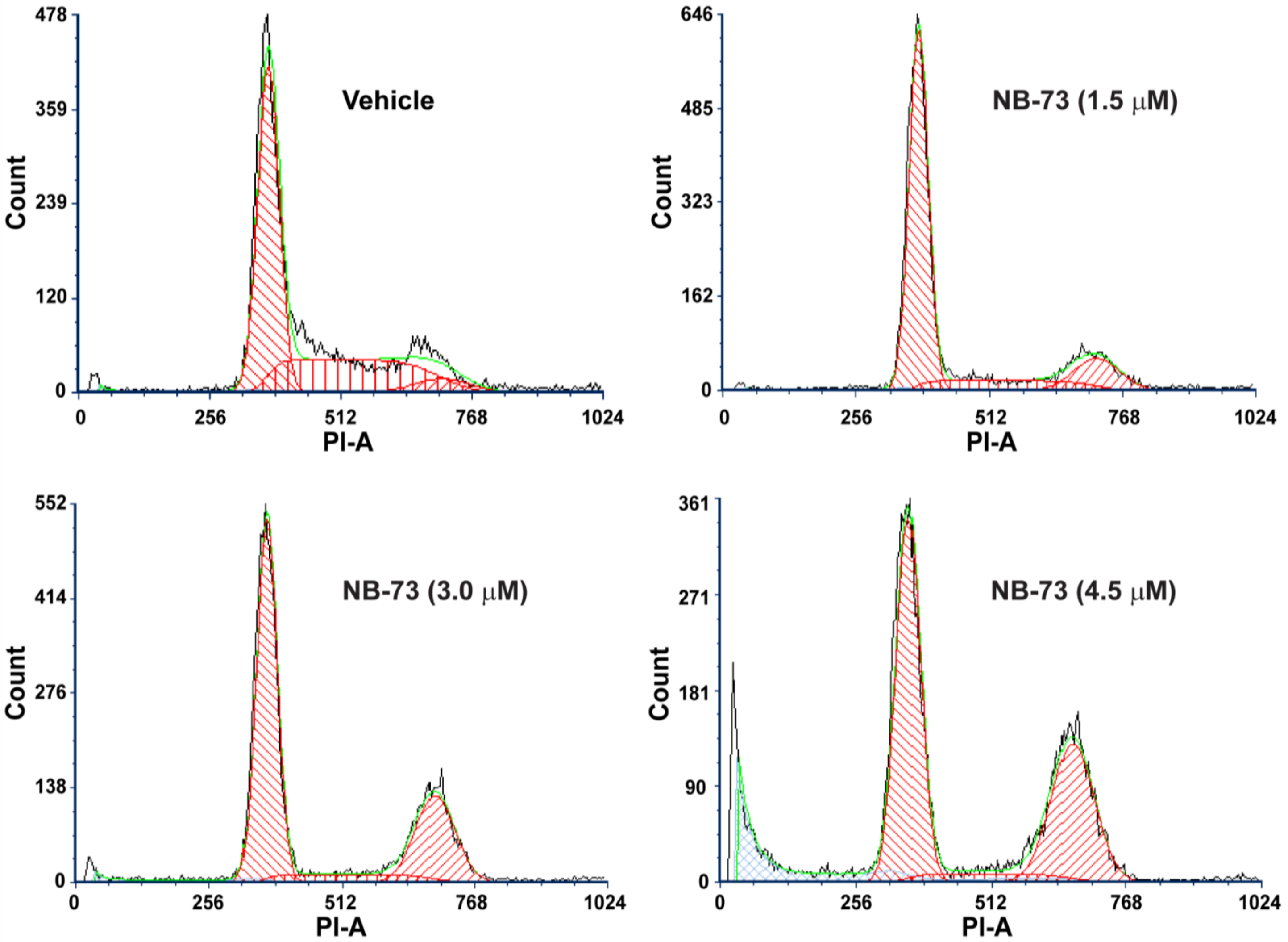

Supplementary Figure 3. Flow Cytometry cell cycle analysis shows that **NB-73** increases the percent of cells in G2/M and reduces the percent of cells in S phase of the cell cycle. MDA-MB-231 cells were synchronized by double thymidine block and were released for 24h with vehicle or **NB-73** treatment (at 1.5, 3.0, or 4.5 µM). Cells were then fixed in 70% ethanol and stained with Propidium Iodide. Cells were then analyzed by Flow Cytometry analyzer BD LSRII for the proportion of cells in different phases of the cell cycle. This figure shows one of the four repeat experiments that were conducted. The summary of findings from all experiments is presented in Figure 4a of the paper.

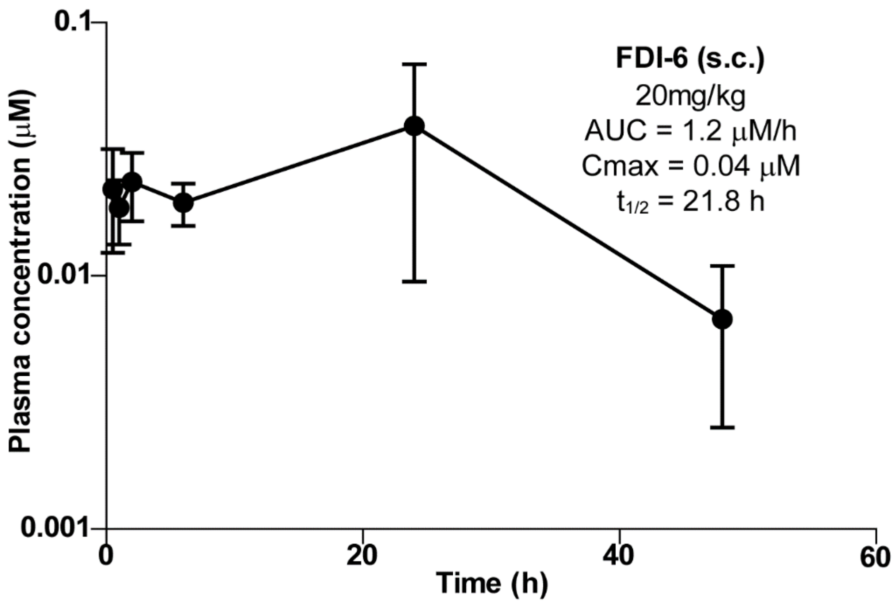

Supplementary Figure 4. Pharmacokinetics and half-life of **FDI-6** in mice. PK was studied after single dose administration via s.c. injection at 20 mg/kg or oral gavage at 40 mg/kg. Multiple plasma samples were collected from each mouse (n=4 for each experiment) over the course of 48 h after **FDI-6** was administered. Compound was quantified using LC-MS/MS. The data were fitted to a non-compartment PK model. **FDI-6** was not detectable in blood after oral administration; therefore, only data from s.c. administration is presented.

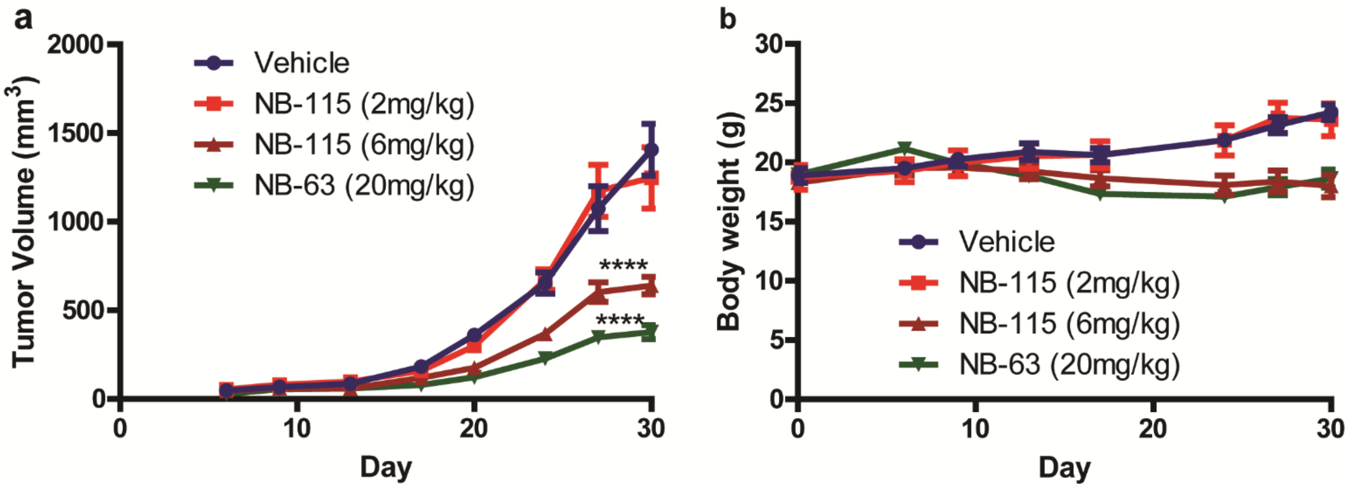

Supplementary Figure 5. Low doses of the compounds **NB-63** (methiodide salt of **NB-55**) and **NB-115** (methiodide salt of **NB-51**) suppress the growth of xenograft tumors. Female NSG animals (8 weeks of age) received $1x10^6$ DT22 cells injected orthotopically into the thoracic mammary gland. **NB-115** was injected s.c. daily at 2 mg/kg or 6 mg/kg, and **NB-63** was injected s.c. daily at 20 mg/kg and then at 10 mg/kg every other day beginning on day 20. (A) Tumor volume and (B) animal body weights were monitored. (2-way ANOVA, Dunnett's post-test; ****, $p<0.0001$, n=8 per group).

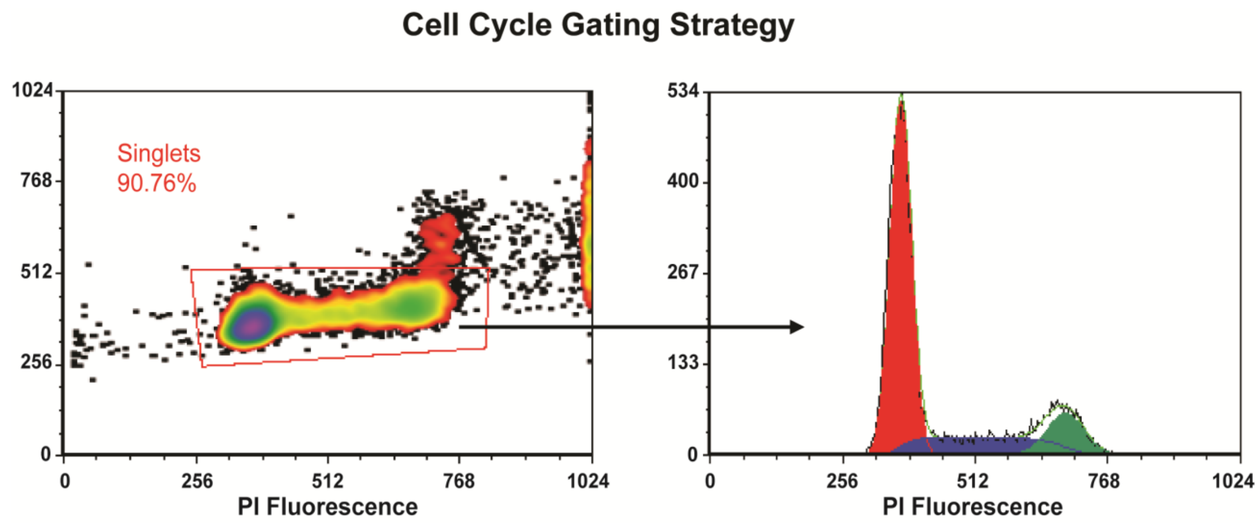

Overlay #	Gate	# of Events	% of gated cells
1	None	11142	100.00
1	Singlets	10113	90.76

Cycle	G1 Mean	G1 CV	% G1	G2 Mean	G2 CV	% G2	% S	G2/G1	% Total
	369.75	5.53	66.76	689.04	5.24	13.79	19.46	1.86	100.00

Supplementary Figure 6. Figure shows the gating strategy used in cell cycle flow cytometry analyses.

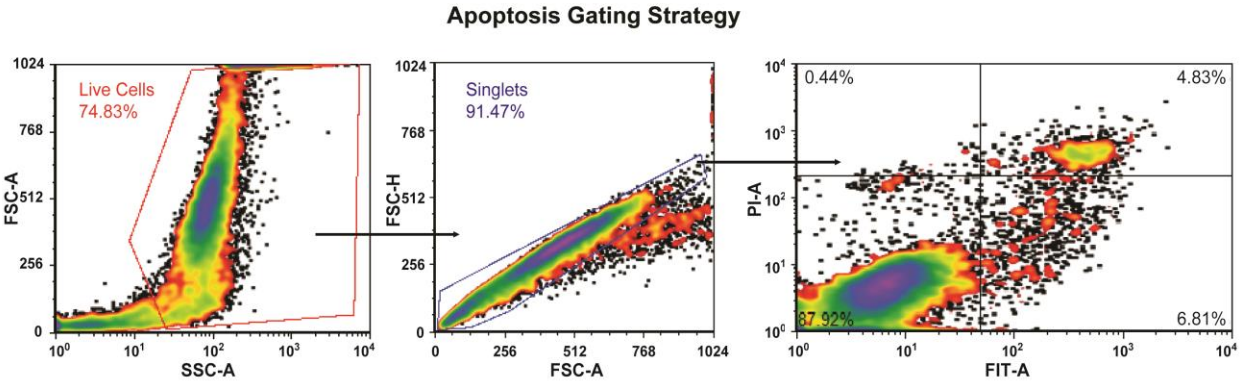

Supplementary Figure 7. Figure shows the gating strategy used in apoptosis flow cytometry analyses.

Supplementary Figure 8. Full uncropped images of blots shown in the paper (Figure 2c, 2d, 2e, 2f, and Figure 4e), including molecular weight markers at the left in kDa when possible**.** We regret we were not able to locate the full blot image for Figure 1b. Predicted molecular weights for FOXM1, FOXA1 and β-Actin are 84 kDa, 49 kDa, and 42 kDa, respectively.

Figure 2c and 2d

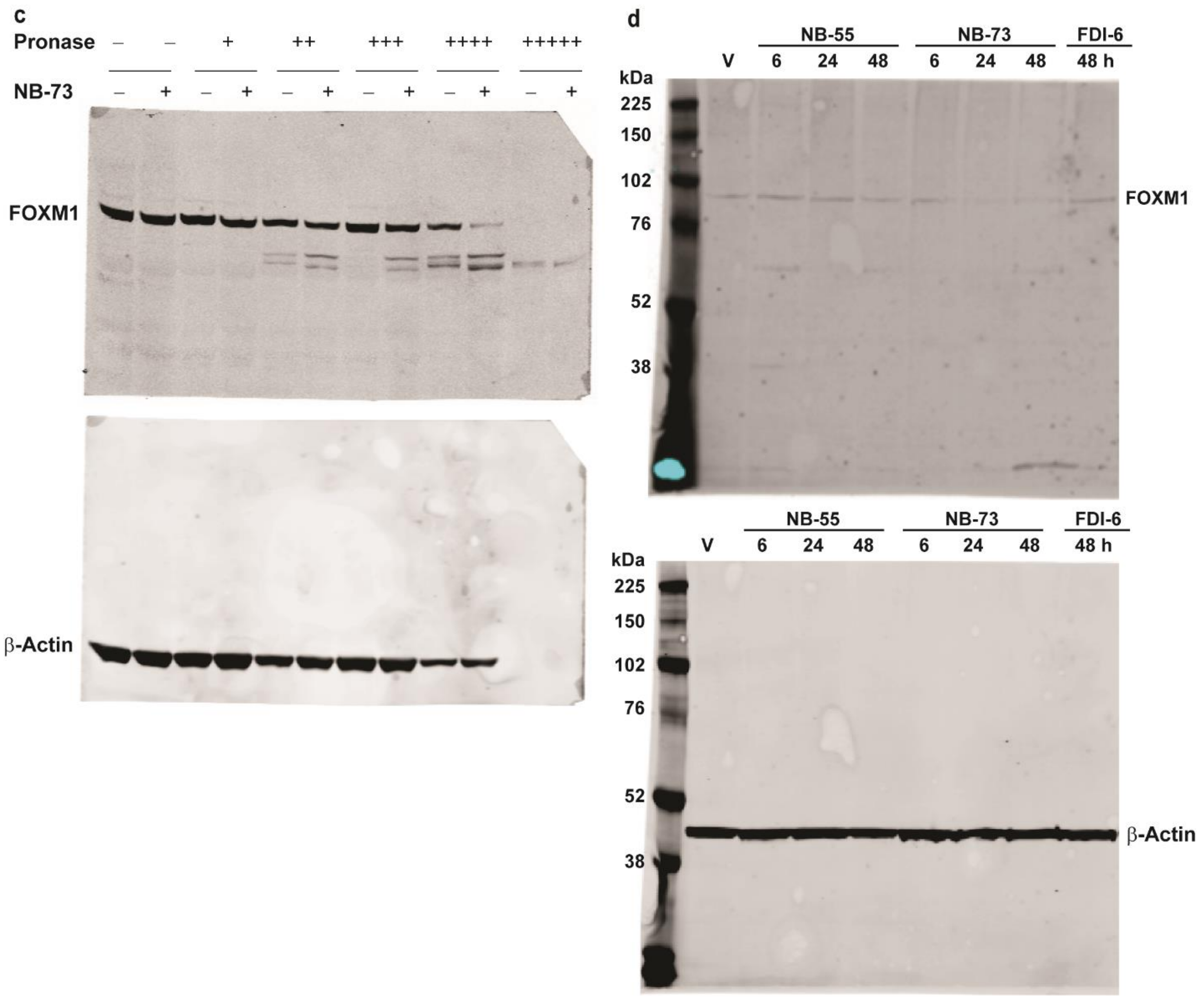

Figure 2e

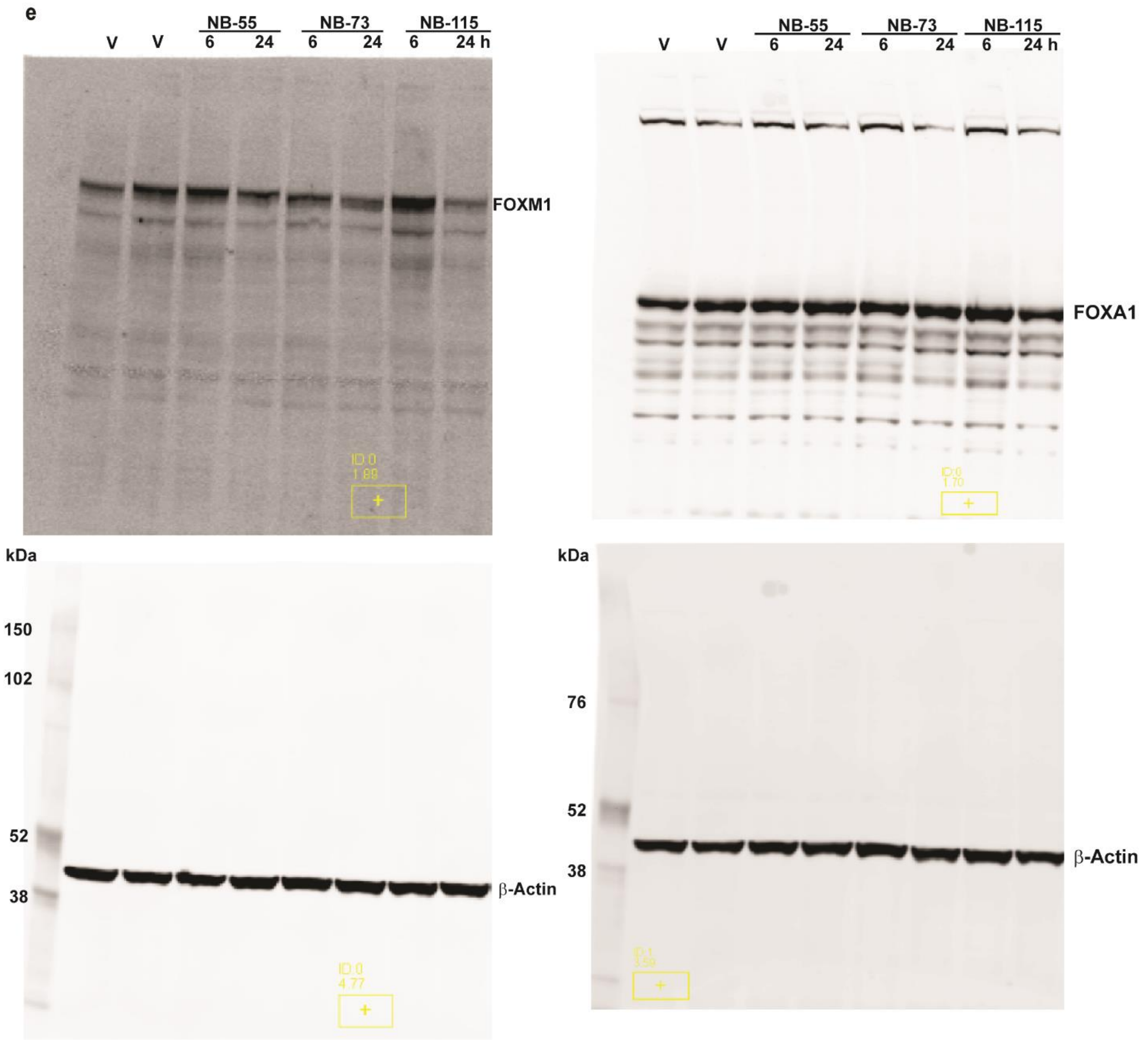
e
NB-55
NB-73
NB-115
V
V
6
24
6
24
6
24 h
FOXM1
FOXA1
kDa
150
102
52
38
76
52
38
β-Actin
β-Actin

Figure 2f

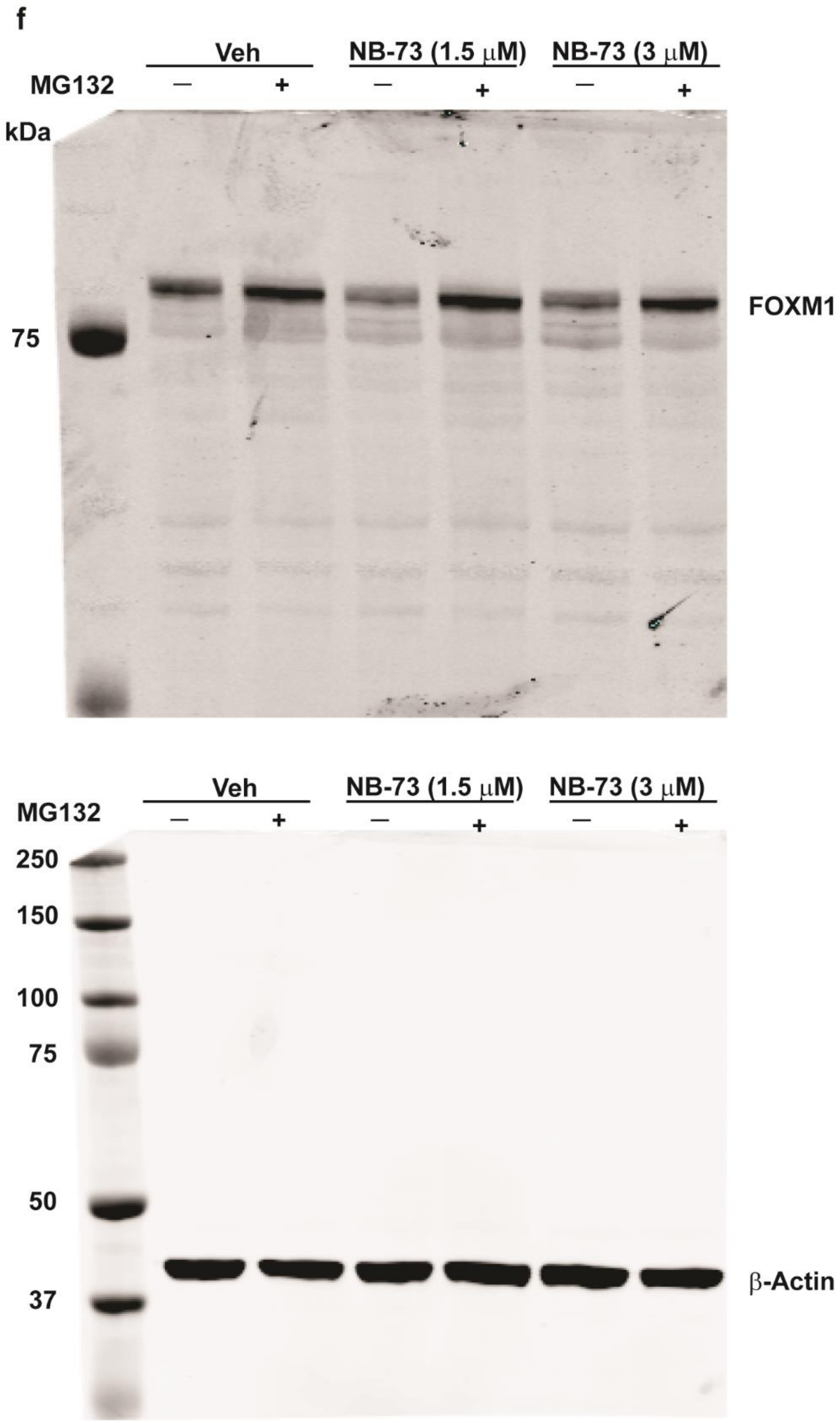
f
Veh
NB-73 (1.5 μM)
NB-73 (3 μM)
MG132
—
+
—
+
—
+
kDa
75
FOXM1
Veh
NB-73 (1.5 μM)
NB-73 (3 μM)
MG132
—
+
—
+
—
+
250
150
100
75
50
37
β-Actin

Figure 4e

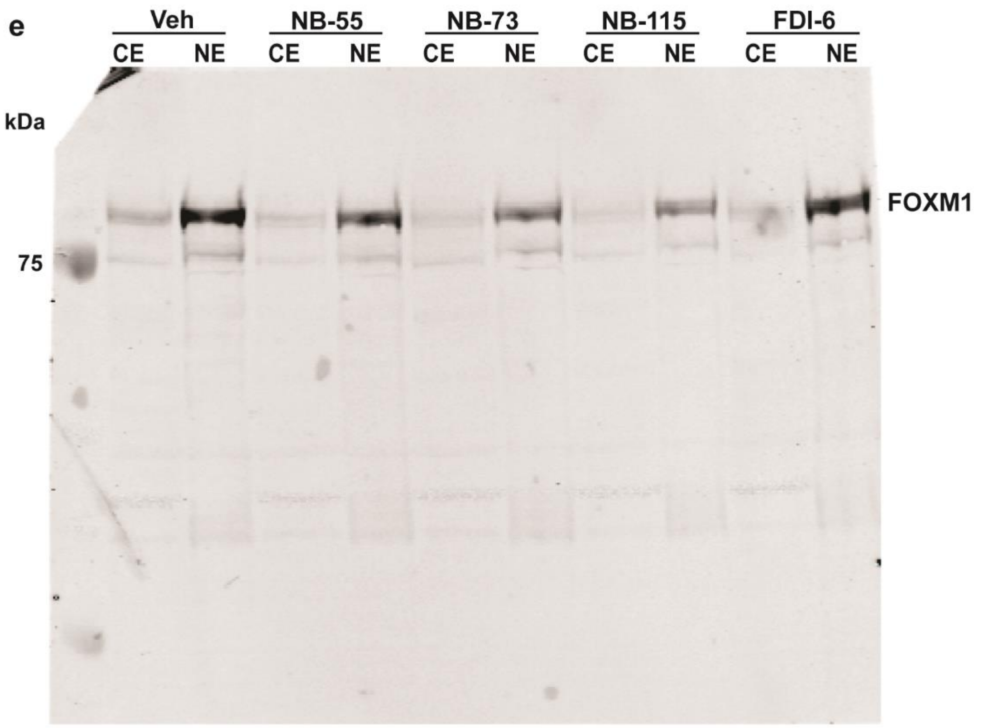
e
Veh
NB-55
NB-73
NB-115
FDI-6
CE
NE
kDa
75
FOXM1

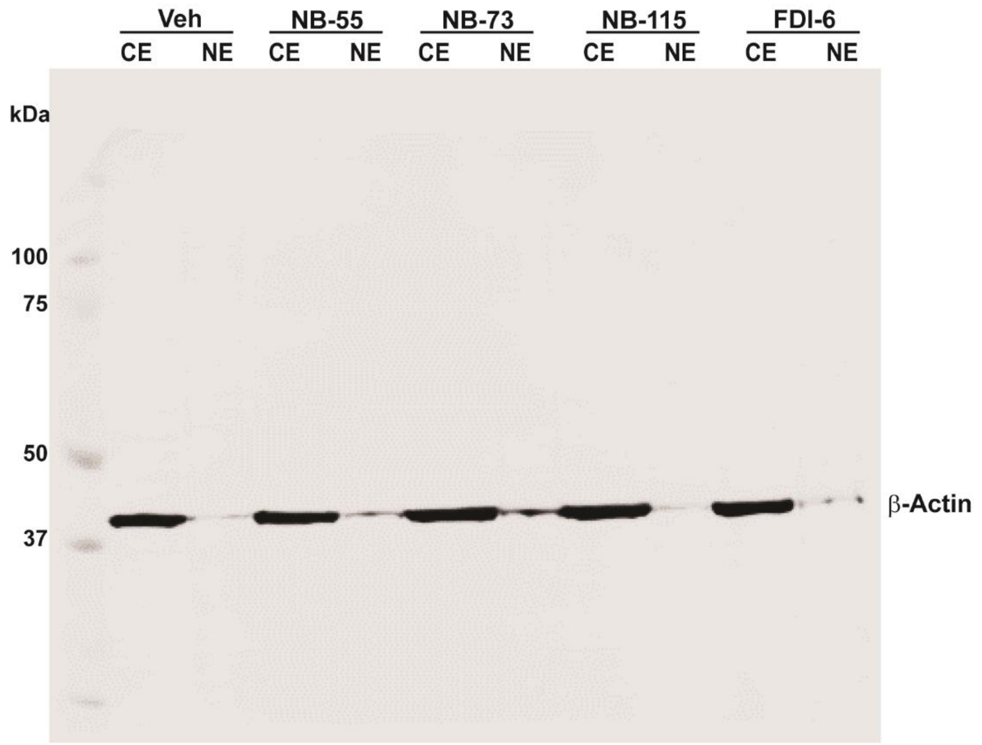
Veh
NB-55
NB-73
NB-115
FDI-6
CE
NE
kDa
100
75
50
37
β-Actin

Supplementary Experimental Methods – Chemical Synthesis

Compounds and materials were supplied from the sources indicated: 4-Chloro-4'-hydroxybenzophnone from Combi-block (CA, San Diego). Pyrrolidine, piperidine, azepane, cesium carbonate, 2-adamantanone, 4,4'-dihydroxybenzophenone, $TiCl_4$, zinc, and 6-bromohexanol: (Aldrich, Milwaukee WI). DMF, methanol, diethyl ether, dichloromethane, methyl iodide, n-hexane, and ethyl acetate were purchased from Fisher science. All solvents were used without further purification. Yields refer to chromatographically and spectroscopically (^{1}H NMR) homogeneous materials, unless otherwise stated. Reactions were monitored by thin layer chromatography (TLC) carried out on Merck silica gel 60 F254 precoated plates (0.25 mm) using UV light as the visualizing agent and ceric ammonium molybdate and heat as developing agents. Preparative TLC plates (silica gel GF, 20 x 20 cm, 1000 micron) were purchased from Analtech Co. Ltd. Flash column chromatography was performed on Silica P Flash silica gel (40-64 μm, 60 Å) from SiliCycle. ^{1}H NMR spectra were recorded at 23 °C on a Varian Unity-400, Varian Inova-500 or Varian Unity-500 spectrometers and are reported in ppm using the residual proton signal as the internal standard ($CHCl_3$, δ = 7.26, center line). The following abbreviations were used to explain the multiplicities: s = singlet, d = doublet, dd = doublet of doublets, t = triplet, q = quartet, m = multiplet, hep = heptet, and br = broad. Proton-decoupled ^{13}C NMR spectra were recorded on a Varian Unity-500 (126 MHz) spectrometer and are reported in ppm using solvent as an internal standard ($CDCl_3$, δ = 77.16, center line). High and low resolution mass spectra were obtained at the University of Illinois Mass Spectrometry Laboratory (Q-TOF Ultima API, Waters Co. Ltd.).

The compounds used in this study were prepared according to the two synthetic routes shown in the schemes below.

Scheme 1. Preparation for NB-55, 118, and 119 and their quaternary ammonium salts

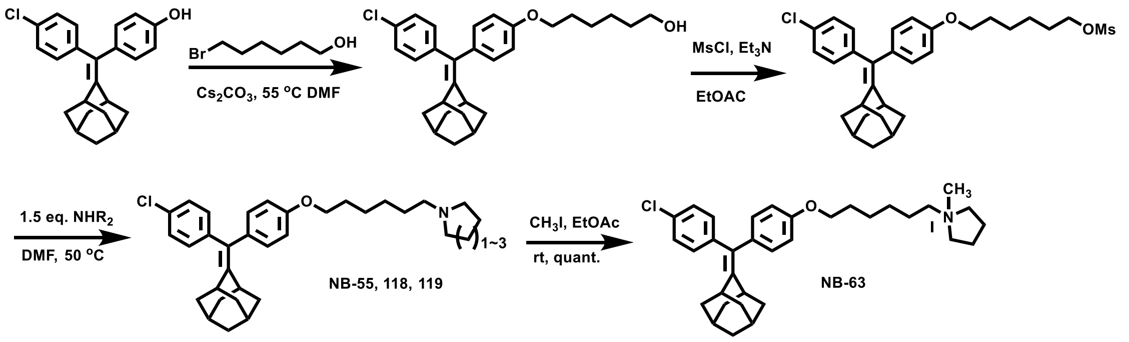

Scheme 2. Preparation for NB-51, 65, 70, and 72 and their quaternary ammonium salts

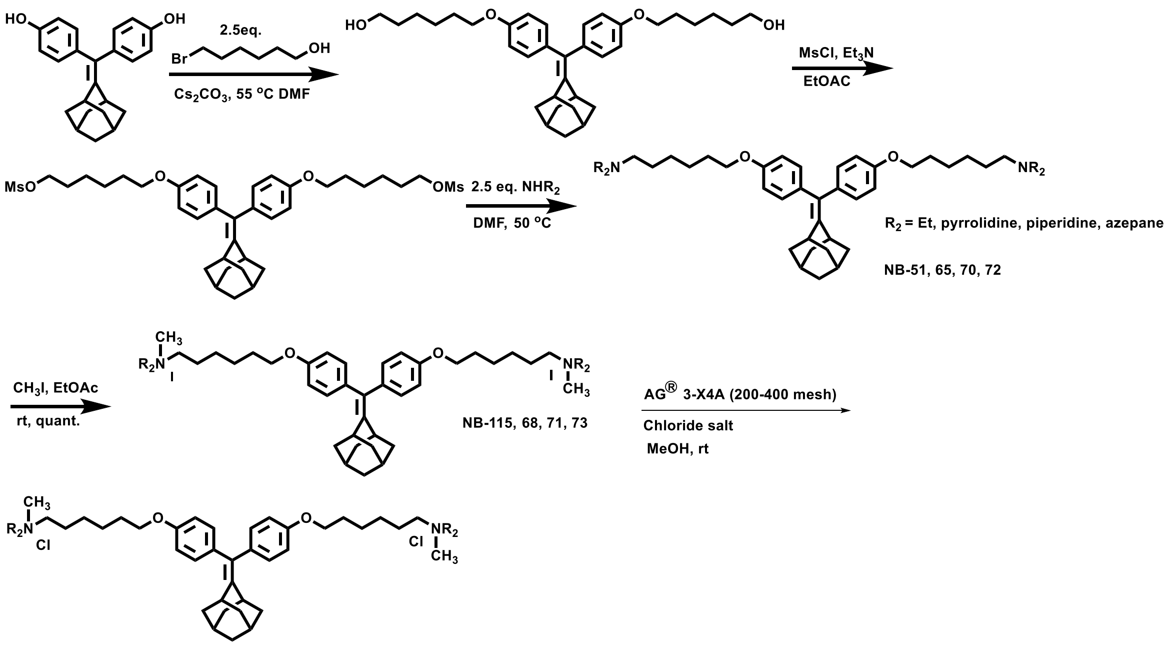

6,6'-(((((5r,7r)-adamantan-2-ylidene)methylene)bis(4,1-phenylene))bis(oxy))bis(hexan-1-ol) (***1***)

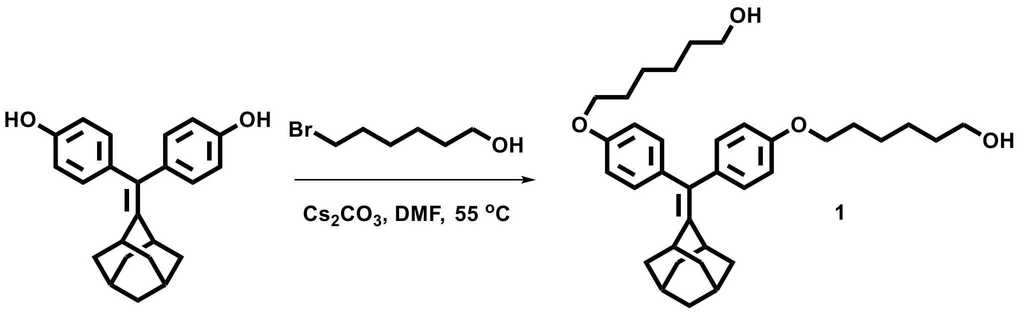

The mixture of 4,4'-(((5r,7r)-adamantan-2-ylidene)methylene)diphenol (332 mg, 1.00 mmol), 6-bromohexanol (434 mg, 2.40 mmol), and cesium carbonate (652 mg, 2.00 mmol) was dissolved and suspended in dried DMF (5 mL) and was heated up and stirred at 55 °C for 5 hr. Once the starting material (bisphenol) disappeared completely by SiO_2 TLC analysis, the reaction was quenched by adding water (50 mL), followed by extraction with ethyl acetate (20 mL x 3), drying over sodium sulfate, concentration under vacuum to load on silica gel column for purification. Elution with a mixture of ethyl acetate and n-hexane (50:50, v/v) provided a sticky semi-solid product (480 mg, 90%).

^{1}H NMR (500 MHz, $CDCl_3$) δ 1.38~1.59 (m, 8H), 1.62 (quintet, *J* = 6.5 Hz, 4H), 1.80 (quintet, *J* = 6.5 Hz, 4H), 1.87 (br s, 10H), 2.01 (s, 2H), 2.81 (s, 2H), 3.67 (t, *J* = 6.5 Hz, 4H), 3.94 (t, *J* = 6.5 Hz, 4H), 6.80 (d, *J* = 8.5 Hz, 4H), 7.03 (d, *J* = 8.5 Hz, 4H). ^{13}C NMR (126 MHz, $CDCl_3$) δ 25.78, 26.19, 28.47, 29.56, 32.94, 34.67, 37.45, 39.85, 63.18, 67.90, 113.99, 129.89, 130.83, 135.92, 145.88, 157.51. HRMS (ESI, M^++1) $C_{35}H_{49}O_4$ Calcd. 533.3631, found 533.3636.

*(((((5r,7r)-Adamantan-2-ylidene)methylene)bis(4,1-phenylene))bis(oxy))bis(hexane-6,1-diyl))dimethanesulfonate (**2**)*

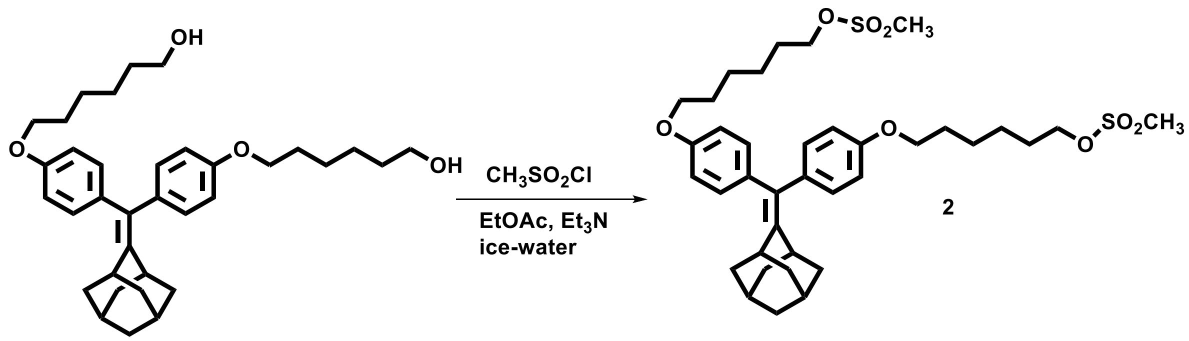

To the mixture of **1** (266 mg, 0.50 mmol) and trimethylamine (303 mg, 0.30 mmol) in ethyl acetate (5 mL) was added dropwise methanesulfonyl chloride (172 mg, 1.50 mmol) at ice-water cooling bath temperature. Once the diol **1** was consumed by SiO_2 TLC analysis, the mixture was

washed by water (5 mL x 3). The ethyl acetate solution was treated with the mixture of $NaHCO_3$ (1 g) and water (5 mL), and was continuously stirred for 1 hr. The ethyl acetate was separated, dried over sodium sulfate, and concentrated under vacuum to afford the title **2** compound as a colorless sticky liquid form (320 mg, 93%). This product was pure enough to be used without further purification.

^{1}H NMR (500 MHz, $CDCl_3$) δ 1.45~1.57 (m, 8H), 1.76~1.83 (m, 8H), 1.87 (br s, 10H), 2.01 (s, 2H), 2.80 (s, 2H), 3.02 (s, 6H), 3.95 (t, J = 6.5 Hz, 4H), 4.26 (t, J = 6.5 Hz, 4H), 6.80 (d, J = 8.5 Hz, 4H), 7.03 (d, J = 8.5 Hz, 4H). ^{13}C NMR (126 MHz, $CDCl_3$) δ 25.49, 25.87, 28.47, 29.33, 29.38, 34.67, 37.44, 37.62, 39.85, 67.68, 70. 22, 113.98, 130.00, 130.84, 135.97, 145.96, 157.44. HRMS (ESI, M^++ Na) $C_{37}H_{52}O_8S_2Na$ Calcd. 711.3001, found 711.3008.

*4-((Z)-((5S,7S)-adamantan-2-ylidene)(4-chlorophenyl)methyl)phenol (**3**)*

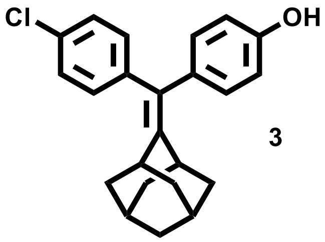

3

To the suspension of zinc powder (392 mg, 6.00 mmol) in THF (20 mL) was added dropwise $TiCl_4$ (567 mg, 3.00 mmol) at -78 °C (dry ice-Acetone), and subsequently the reaction mixture was refluxed for 2 hr and then cooled down to room temperature before addition of a mixture of 2-adamantanone (155 mg, 1.03 mmol) and 4-chloro-4'-hydroxybenzophenone (236 mg, 1.00 mmol) in THF (10 mL). The reaction mixture was refluxed again for 3 hr. The reaction mixture was cooled to room temperature, poured into a sat. $NaHCO_3$ aqueous solution, and stirred until the suspension color changed from black to white. The mixture was extracted with EtOAc (20 mL x 3), washed with brine, and dried over Na_2SO_4. The solvent was evaporated under vacuum,

followed by a column chromatography on SiO_2 using 10% EtOAc/n-Hexane as an eluent to afford title compound (280 mg, 80%).

^{1}H NMR (500 MHz, $CDCl_3$) δ 1.80~1.97 (m, 10H), 2.02 (s, 2H), 2.74 (s, 1H), 2.82 (s, 1H), 6.76 (d, *J* = 8.5 Hz, 2H), 6.99 (d, *J* = 8.5 Hz, 2H), 7.06 (d, *J* = 8.5 Hz, 2H), 7.27 (d, *J* = 8.5 Hz, 2H). ^{13}C NMR (126 MHz, $CDCl_3$) δ 28.36, 31.26, 34.65, 34.74, 36.75, 37.32, 39.79, 115.14, 128.35, 129.14, 131.06, 131.15, 131.94, 135.38, 141.91, 147.36, 154.18. HRMS (EI, M^+) $C_{23}H_{23}OCl$ Calcd. 350.14375, found 350.14366.

*6-(4-((Z)-((5S,7S)-adamantan-2-ylidene)(4-chlorophenyl)methyl)phenoxy)hexan-1-ol (**4**)*

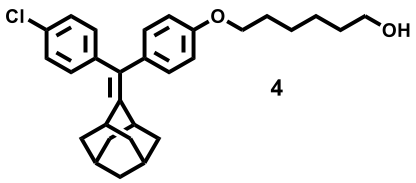

4

The title compound (**4**) was prepared from the reaction of the compound **3** (175 mg, 0.50 mmol) with 6-bromohexanol (271 mg, 1.50 mmol), and cesium carbonate (326 mg, 1.00 mmol) in 93% yield, following the same procedure described for the preparation of the compound **1** (Bisphenoxyhexanol).

^{1}H NMR (500 MHz, $CDCl_3$) δ 1.40~1.55 (m, 4H), 1.62 (quintet, *J* = 7.0 Hz, 2H), 1.80 (quintet, *J* = 7.0 Hz, 2H), 1.85~1.92 (m, 10H), 2.02 (s, 2H), 2.74 (s, 1H), 2.82 (s, 1H), 3.68 (t, *J* = 7.0 Hz, 2H), 3.95 (t, *J* = 7.0 Hz, 2H), 6.81 (d, *J* = 8.5 Hz, 2H), 7.02 (d, *J* = 8.5 Hz, 2H), 7.06 (d, *J* = 8.5 Hz, 2H), 7.24 (d, *J* = 8.5 Hz, 2H). ^{13}C NMR (126 MHz, $CDCl_3$) δ 25.78, 26.18, 28.36, 29.54, 32.94, 34.65, 37.33, 39.80, 55.13, 63.18, 67.94, 114.15, 128.32, 129.26, 130.84, 131.16, 131.89, 135.06, 141.98, 147.21, 157.74. HRMS (EI, M^++1) $C_{29}H_{36}OCl$ Calcd. 451.2404, found 451.2399.

*6-(4-((Z)-((5S,7S)-adamantan-2-ylidene)(4-chlorophenyl)methyl)phenoxy)hexyl methanesulfonate (**5**)*

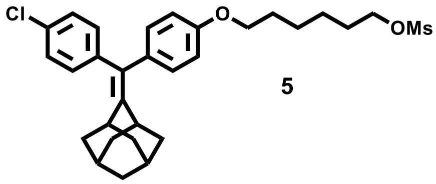

5

The title compound (**5**) was prepared from the reaction of the compound **4** (135 mg, 0.30 mmol) with methanesulfonyl chloride (51 mg, 0.45 mmol), and triethylamine (101 mg, 1.00 mmol) in 98% yield, following the same procedure described for the preparation of the compound **2**.

^{1}H NMR (500 MHz, $CDCl_3$) δ 1.48~1.58 (m, 4H), 1.83 (quintet, *J* = 7.0 Hz, 4H), 1.84~1.91 (m, 10H), 2.02 (s, 2H), 2.74 (s, 1H), 2.82 (s, 1H), 3.02 (s, 3H), 3.95 (t, *J* = 6.5 Hz, 2H), 4.26 (t, *J* = 6.5 Hz, 2H), 6.81 (d, *J* = 8.5 Hz, 2H), 7.02 (d, *J* = 8.5 Hz, 2H), 7.06 (d, *J* = 8.5 Hz, 2H), 7.24 (d, *J* = 8.5 Hz, 2H). ^{13}C NMR (126 MHz, $CDCl_3$) δ 25.49, 25.87, 28.36, 29.33, 29.37, 34.66, 37.74, 37.32, 37.62, 39.80, 67.72, 70.22, 114.13, 128.33, 129.22, 130.86, 131.16, 131.90, 135.13, 141.97, 147.25, 157.66. HRMS (ESI, M^++1) $C_{30}H_{38}O_4SCl$ Calcd. 529.2179, found 529.2183.

*1-(6-(4-((Z)-((5S,7S)-adamantan-2-ylidene)(4-chlorophenyl)methyl)phenoxy)hexyl)pyrrolidine **(NB-55)***

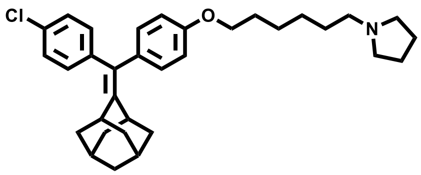

The mixture of the compound **5** (53 mg, 0.10 mmol) and pyrrolidine (28 mg, 0.40 mmol) in DMF (100 μL) was stirred at 55 °C for 3 hr. The solvent was evaporated under a gentle stream of nitrogen. To this was added $NaHCO_3$ (40 mg) and deionized water (1 mL), and product was

extracted with EtOAc (400 μL x 3). The extract was washed with brine, water, and dried over Na_2SO_4 to provide **NB-55** (48 mg) as a sticky liquid.

^1H NMR (500 MHz, $CDCl_3$) δ 1.41 (quintet, *J* = 7.0 Hz, 2H), 1.48 (quintet, *J* = 7.0 Hz, 2H), 1.56 (quintet, *J* = 7.0 Hz, 2H), 1.76~1.81 (m, 6H), 1.82~1.92 (m, 10H), 2.01 (s, 2H), 2.44 (t, *J* = 6.5 Hz, 2H), 2.46~2.51 (m, 4H), 2.74 (s, 1H), 2.82 (s, 1H), 3.93 (t, *J* = 6.5 Hz, 2H), 6.81 (d, *J* = 8.5 Hz, 2H), 7.02 (d, *J* = 8.5 Hz, 2H), 7.06 (d, *J* = 8.5 Hz, 2H), 7.24 (d, *J* = 8.5 Hz, 2H). ^{13}C NMR (126 MHz, $CDCl_3$) δ 23.62, 26.34, 27.76, 28.36, 29.27, 29.54, 34.64, 34.73, 37.32, 39.80, 54.48, 56.86, 68.01, 114.15, 128.32, 129.27, 130.83, 131.17, 131.87, 135.00, 141.99, 147.18, 157.76. HRMS (ESI, M^++1) $C_{33}H_{43}ONCl$ Calcd. 504.3033, found 504.3033.

*1-(6-(4-((Z)-((5S,7S)-adamantan-2-ylidene)(4-chlorophenyl)methyl)phenoxy)hexyl)-1-methyl-1λ^4-pyrrolidine (**NB-63**)*

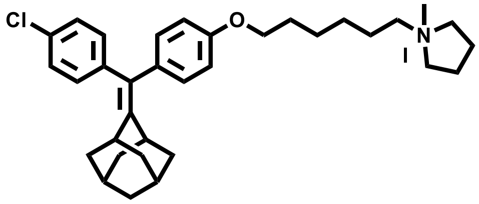

The solution of **NB-55** (60 mg, 0.12 mmol) and methyl iodide (200 μL) in ethyl acetate (500 μL) was heated at 50 °C for 1 hr to generate a precipitate. The evaporation of solvent afforded **NB-63** quantitatively. This is pure enough to use without further purification.

^1H NMR (500 MHz, $CDCl_3$ + CD_3OD) δ 1.45~1.63 (m, 4H), 1.77~1.93 (m, 14H), 2.02 (s, 2H), 2.23~2.40 (br s, 4H), 2.73 (s, 1H), 2.80 (s, 1H), 3.28 (s, 3H), 3.69 (t, *J* = 7.0 Hz, 2H), 3.82 (br s, 4H), 3.95 (t, *J* = 7.0 Hz, 2H), 6.82 (d, *J* = 8.5 Hz, 2H), 7.02 (d, *J* = 8.5 Hz, 2H), 7.07 (d, *J* = 8.5 Hz, 2H), 7.25 (d, *J* = 8.5 Hz, 2H).). ^{13}C NMR (126 MHz, $CDCl_3$ + CD_3OD) δ 21.97, 23.66, 24.31, 26.02, 26.28, 28.35, 29.24, 34.65, 37.13, 39.80, 53.95, 64.56, 65.06, 67.61.

HRMS (ESI, M^+) $C_{34}H_{45}ONCl$ Calcd. 518.3190, found 518.3181.

*1-(6-(4-((Z)-((5S,7S)-Adamantan-2-ylidene)(4-chlorophenyl)methyl)phenoxy)hexyl)piperidine (**NB-118**)*

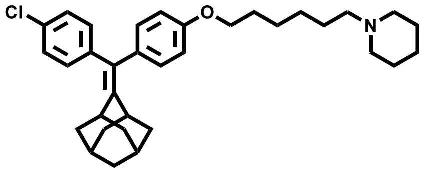

NB-118 (44 mg, sticky liquid) was obtained from the reaction of the compound **5** (50 mg, 0.095 mmol) and piperidine (45 mg, 0.53 mmol) in 89% yield, as described for the preparation of **NB-55**.

^{1}H NMR (500 MHz, $CDCl_3$) δ 1.31 (quintet, *J* = 7.0 Hz, 2H), 1.36 (quintet, *J* = 7.0 Hz, 2H), 1.40~1.56 (m, 4H), 1.56~1.63 (m, 4H), 1.78 (quintet, *J* = 7.0 Hz, 2H), 1.83~1.91 (m, 10H), 2.01 (s, 2H), 2.29 (t, *J* = 6.5 Hz, 2H), 2.32~2.44 (m, 4H), 2.74 (s, 1H), 2.82 (s, 1H), 3.92 (t, *J* = 6.5 Hz, 2H), 6.81 (d, *J* = 8.5 Hz, 2H), 7.01 (d, *J* = 8.5 Hz, 2H), 7.06 (d, *J* = 8.5 Hz, 2H), 7.24 (d, *J* = 8.5 Hz, 2H). ^{13}C NMR (126 MHz, $CDCl_3$) δ 24.76, 26.33, 27.78, 28.03, 28.36, 29.26, 34.64, 37.33, 39.80, 54.92, 68.01, 114.15, 128.32, 129.28, 130.82, 131.16, 131.16, 131.88, 135.00, 141.99, 147.18, 157.78. HRMS (ESI, M^++1) $C_{34}H_{45}ONCl$ Calcd. 518.3190, found 518.3192.

*1-(6-(4-((Z)-((5S,7S)-adamantan-2-ylidene)(4-chlorophenyl)methyl)phenoxy)hexyl)azepane (**NB-119**)*

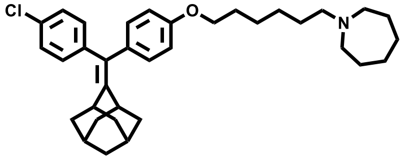

NB-118 (44 mg, sticky liquid) was obtained from the reaction of the compound **5** (50 mg, 0.095 mmol) and azepane (40 mg, 0.40 mmol) in 87% yield, as described for the preparation of **NB-55**.

^{1}H NMR (500 MHz, $CDCl_3$) δ 1.31 (quintet, *J* = 7.0 Hz, 2H), 1.35 (quintet, *J* = 7.0 Hz, 2H), 1.44~1.55 (m, 4H), 1.56~1.76 (m, 6H), 1.77 (quintet, *J* = 7.0 Hz, 2H), 1.83~1.91 (m, 10H), 2.00 (s, 2H), 2.44 (t, *J* = 6.5 Hz, 2H), 2.62 (t, *J* = 6.0 Hz, 4H), 2.73 (s, 1H), 2.81 (s, 1H), 3.93 (t, *J* = 6.5 Hz, 2H), 6.80 (d, *J* = 8.5 Hz, 2H), 7.00 (d, *J* = 8.5 Hz, 2H), 7.05 (d, *J* = 8.5 Hz, 2H), 7.23 (d, *J* = 8.5 Hz, 2H). ^{13}C NMR (126 MHz, $CDCl_3$) δ 26.33, 27.26, 27.65, 27.75, 28.18, 28.37, 29.56, 34.64, 34.74, 37.33, 55.84, 58.51, 68.01, 114.14, 128.30, 129.27, 130.81, 131.15, 131.87, 134.98, 141.98, 147.16, 157.77. HRMS (ESI, M^++1) $C_{35}H_{47}ONCl$ Calcd. 532.3346, found 532.3347.

*1,1'-((((((5r,7r)-adamantan-2-ylidene)methylene)bis(4,1-phenylene))bis(oxy))bis(hexane-6,1-diyl))dipyrrolidine (**NB-65**)*

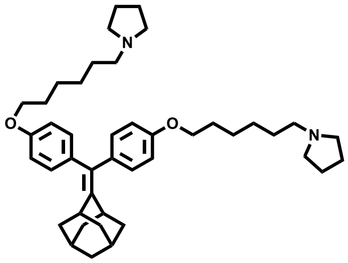

The mixture of the compound **2** (68 mg, 0.10 mmol) and pyrrolidine (57 mg, 0.80 mmol) in DMF (200 μL) was stirred at 55 °C for 3 hr. The solvent was evaporated under a gentle stream of nitrogen. To this was added $NaHCO_3$ (80 mg) and deionized water (1 mL), and product was extracted with EtOAc (400 μL x 3). The extract was washed with brine, water, and dried over Na_2SO_4 to provide **NB-65** (51 mg) as a sticky liquid.

^{1}H NMR (500 MHz, $CDCl_3$) δ 1.40 (quintet, J = 7.0 Hz, 4H), 1.49 (quintet, J = 7.0 Hz, 4H), 1.56 (quintet, J = 7.0 Hz, 4H), 1.74~1.82 (m, 12H), 1.82~1.92 (m, 10H), 2.01 (s, 2H), 2.43 (t, J = 7.0 Hz, 4H), 2.47~2.55 (m, 8H), 2.81 (s, 2H), 3.93 (t, J = 6.0 Hz, 4H), 6.80 (d, J = 8.5 Hz, 4H), 7.03 (d, J = 8.5 Hz, 4H). ^{13}C NMR (126 MHz, $CDCl_3$) δ 23.66, 26.37, 27.79, 28.50, 29.35, 29.58, 34.68, 37.48, 39.87, 54.54, 56.90, 68.01, 114.01, 129.95, 130.82, 135.89, 145.83, 157.56.

HRMS (ESI, M^{+}+1) $C_{43}H_{63}O_2N_2$ Calcd. 639.4890, found 639.4909.

*1,1'-((((((5r,7r)-adamantan-2-ylidene)methylene)bis(4,1-phenylene))bis(oxy))bis(hexane-6,1-diyl))bis(1-methyl-1λ^4-pyrrolidine) (**NB-68**)*

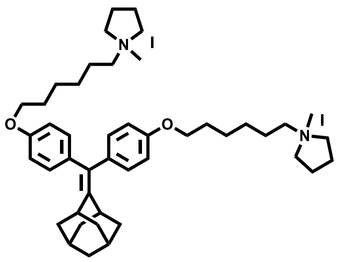

The mixture of **NB-65** (32 mg, 0.05 mmol) and methyl iodide (100 μL) in ethyl acetate (500 μL) was heated up at 50 °C for 1 hr to form a precipitate. The evaporation of solvent afforded **NB-68** (45 mg) quantitatively in a form that was pure enough to use without further purification.

^{1}H NMR (500 MHz, $CDCl_3$ + CD_3OD) δ 1.38 (quintet, J = 7.5 Hz, 4H), 1.47 (quintet, J = 7.5 Hz, 4H), 1.59~1.82 (m, 18H), 1.89 (s, 2H), 2.07~2.25 (m, 8H), 2.66 (s, 2H), 3.03 (s, 6H), 3.38 (t, J = 7.5 Hz, 4H), 3.54 n(s, 8H), 3.86 (t, t, J = 7.5 Hz, 4H), 6.69 (d, J = 8.5 Hz, 4H), 6.91 (d, J = 8.5 Hz, 4H). ^{13}C NMR (126 MHz, $CDCl_3$ + CD_3OD) δ 14.15, 21.77, 24.02, 25.76, 26.09, 28.31, 29.00, 34.52, 37.25, 39.67, 64.85, 67.55, 113.98, 129.58, 130.64, 135.99, 146.02, 157.17.

HRMS (ESI, M^{2+}) $C_{45}H_{66}O_2N_2$ Calcd. 334.2640, found 334.2643.

*1,1'-((((((5r,7r)-Adamantan-2-ylidene)methylene)bis(4,1-phenylene))bis(oxy))bis(hexane-6,1-diyl))dipiperidine (**NB-70**)*

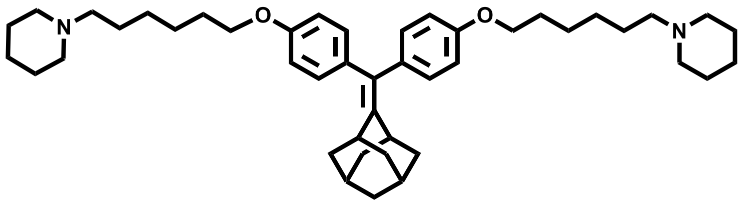

NB-70 (36 mg, sticky liquid) was obtained from the reaction of the compound **2** (50 mg, 0.058 mmol) and piperidine (45 mg, 0.53 mmol) in 92% yield, as described for the preparation of **NB-65**.

1H NMR (500 MHz, $CDCl_3$) δ 1.36 (quintet, J = 7.0 Hz, 4H), 1.40~1.58 (m, 12H), 1.59 (quintet, J = 7.0 Hz, 8H), 1.78 (quintet, J = 7.0 Hz, 4H), 1.82~1.92 (m, 10H), 2.00 (s, 2H), 2.30 (t, J = 7.0 Hz, 4H), 2.32~2.44 (br s, 8H), 2.81 (s, 2H), 3.93 (t, J = 6.5 Hz, 4H), 6.80 (d, *J* = 8.5 Hz, 4H), 7.03 (d, *J* = 8.5 Hz, 4H). ^{13}C NMR (126 MHz, $CDCl_3$) δ 24.77, 26.28, 26.36, 27.18, 27.81, 28.49, 29.56, 34.66, 37.46, 39.86, 54.94, 59.85, 67.96, 113.99, 129.94, 130.81, 135.87, 145.81, 157.54. HRMS (ESI, M^++1) $C_{45}H_{67}O_2N_2$ Calcd. 667.5203, found 667.5210.

*1,1'-((((((5r,7r)-Adamantan-2-ylidene)methylene)bis(4,1-phenylene))bis(oxy))bis(hexane-6,1-diyl))bis(1-methyl-1λ^4-piperidine) diodide (**NB-71**)*

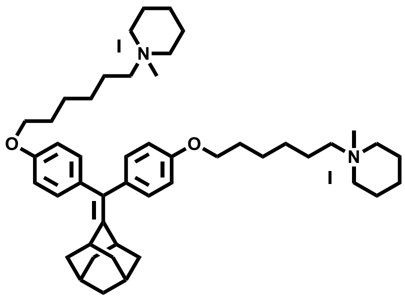

NB-71 (70 mg) was prepared from the reaction of the **NB-70** (40 mg, 0.075 mmol) and methyl iodide (200 µL) in quantitative yield by following the procedure as described for the preparation of **NB-68**.

^{1}H NMR (500 MHz, $CDCl_3$ + CD_3OD) δ 1.43 (quintet, *J* = 7.5 Hz, 4H), 1.52 (quintet, *J* = 7.5 Hz, 4H), 1.63~1.92 (m, 30H), 1.94 (s, 2H), 2.71 (s, 2H), 3.13 (s, 6H), 3.43~3.54 (m, 12H), 3.91 (t, *J* = 7.5 Hz, 4H), 6.75 (d, *J* = 8.5 Hz, 4H), 6.96 (d, *J* = 8.5 Hz, 4H). ^{13}C NMR (126 MHz, $CDCl_3$ + CD_3OD) δ 20.29, 20.87, 22.10, 25.84, 26.11, 28.36, 29.08, 34.57, 37.33, 39.74, 48.50, 61.47, 63.89, 67.61, 114.06, 129.60, 130.70, 136.02, 146.08, 157.23. HRMS (ESI, M^{2+}) $C_{47}H_{70}O_2N_2$ Calcd. 348.2797, found 348.2798.

*1,1'-((((((5r,7r)-Adamantan-2-ylidene)methylene)bis(4,1-phenylene))bis(oxy))bis(hexane-6,1-diyl))bis(azepane) (**NB-72**)*

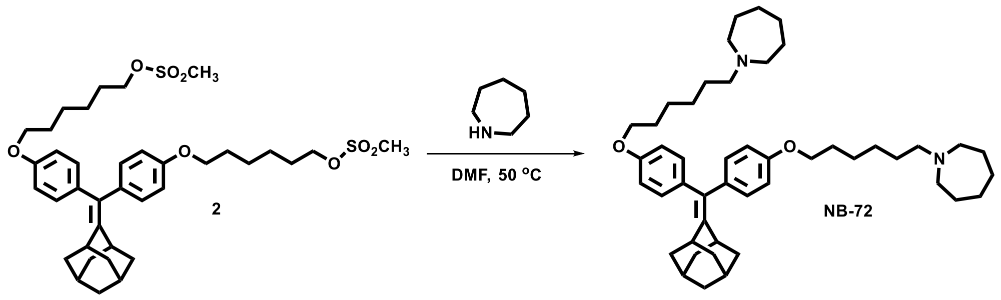

NB-72 (37 mg, sticky liquid) was obtained from the reaction of the compound **2** (50 mg, 0.058 mmol) and azepane (55 mg, 0.55 mmol) in 91% yield, following the procedure as described for the preparation of **NB-65**.

^{1}H NMR (500 MHz, $CDCl_3$) δ 1.35 (quintet, *J* = 7.5 Hz, 4H), 1.49 (quintet, *J* = 7.5 Hz, 4H), 1.51 (quintet, *J* = 7.5 Hz, 4H), 1.61 (br s, 8H), 1.65 (br s, 8H), 1.77 (quintet, *J* = 7.5 Hz, 4H) , 1.86 (br s, 10H), 2.01 (s, 2H), 2.47 (t, *J* = 7.5 Hz, 4H), 2.63 (t, *J* = 6.0 Hz, 8H), 2.81 (s, 2H), 3.93 t, *J* =

6.0 Hz, 4H), 6.80 (d, *J* = 8.5 Hz, 4H), 7.03 (d, *J* = 8.5 Hz, 4H). ^{13}C NMR (126 MHz, $CDCl_3$) δ 26.35, 27.26, 27.66, 27.76, 28.21, 28.48, 29.59, 34.66, 37.46, 39.86, 55.85, 58.53, 67.99, 113.98, 125.22, 130.82, 135.87, 145.82, 157.55. HRMS (ESI, M^++1) $C_{47}H_{71}O_2N_2$ Calcd. 695.5516, found 695.5529.

*1,1'-((((((5r,7r)-adamantan-2-ylidene)methylene)bis(4,1-phenylene))bis(oxy))bis(hexane-6,1-diyl))bis(1-methyl-1λ^4-azepane) diiodide (**NB-73**)*

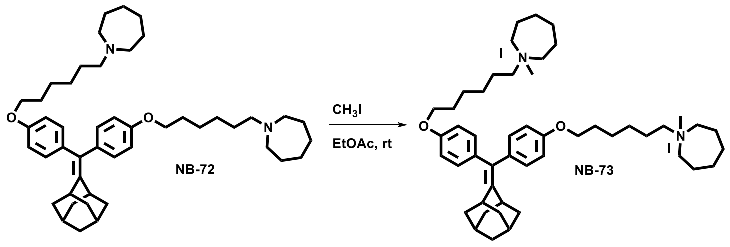

NB-73 (47 mg) was prepared from the reaction of the **NB-72** (35 mg, 0.05 mmol) and methyl iodide (100 µL) quantitatively by following the procedure described for the preparation of **NB-68**.

^{1}H NMR (500 MHz, $CDCl_3$ + CD_3OD) δ 1.43 (quintet, *J* = 7.5 Hz, 4H), 1.55 (quintet, *J* = 7.5 Hz, 4H), 1.71 (br s, 8H), 1.73~1.92 (m, 26H), 1.95 (s, 2H), 2.73 (s, 2H), 3.07 (s, 6H), 3.27~3.50 (m, 12H), 3.94 (t, *J* = 7.5 Hz, 4H), 6.76 (d, *J* = 8.5 Hz, 4H), 6.98 (d, *J* = 8.5 Hz, 4H). ^{13}C NMR (126 MHz, $CDCl_3$ + CD_3OD) δ 21.84, 22.69, 25.83, 26.20, 27.74, 28.40, 29.12, 34.58, 37.26, 39.65, 50.45, 64.76, 65.44, 67.58. 113.94, 129.88, 130.62, 135.94, 145.87, 157.36. HRMS (ESI, M^{2+}) $C_{49}H_{76}O_2N_2$ Calcd. 363.3032, found 363.2957.

*6,6'-(((((5r,7r)-adamantan-2-ylidene)methylene)bis(4,1-phenylene))bis(oxy))bis(N,N-diethylhexan-1-amine) (**NB-51**)*

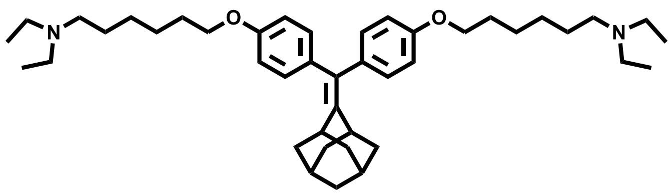

NB-51 (33 mg) was obtained from the reaction of the compound **2** (50 mg, 0.058 mmol) and diethyl amine (50 mg, 0.68 mmol) in 89% yield, following the procedure described for the preparation of **NB-65**.

^{1}H NMR (500 MHz, $CDCl_3$) δ 1.03 (t, *J* = 7.0 Hz, 12H), 1.37 (quintet, *J* = 7.0 Hz, 4H), 1.42~1.52 (m, 8H), 1.79 (quintet, *J* = 7.0 Hz, 4H), 1.82~1.92 (m, 10H), 2.00 (br s, 2H), 2.43 (t, *J* = 7.5 Hz, 4H), 2.53 (q, *J* = 7.0 Hz, 8H), 2.81 (br s, 2H), 3.93 (t, *J* = 6.0 Hz, 2H), 6.80 (d, *J* = 8.5 Hz, 4H), 7.02 (d, *J* = 8.5 Hz, 4H). ^{13}C NMR (126 MHz, $CDCl_3$) δ 11.91, 26.36, 27.18, 27.75, 28.48, 29.60, 34.66, 37.46, 39.85, 47.13, 53.12, 67.97, 113.98, 129.93, 130.81, 135.87, 145.82, 157.54. HRMS (ESI, M^+) $C_{43}H_{67}O_2N_2$ Calcd. 643.5203, found 643.5225.

*((((((5r,7r)-Adamantan-2-ylidene)methylene)bis(4,1-phenylene))bis(oxy))bis(hexane-6,1-diyl))bis(diethyl(methyl)-λ^4-azane) diiodide (**NB-115**)*

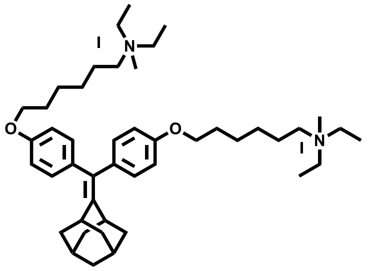

NB-115 (45 mg) was prepared from the reaction of the **NB-51** (32 mg, 0.05 mmol) and methyl iodide (100 μL) quantitatively by following the procedure described for the preparation of **NB-68**.

^{1}H NMR (500 MHz, $CDCl_3$) δ 1.32 (t, *J* = 7.0 Hz, 12H), 1.45 (quintet, *J* = 7.0 Hz, 4H), 1.53 (quintet, *J* = 7.0 Hz, 4H), 1.64~1.76 (m, 8H), 1.76~1.85 (m, 10H), 1.96 (br s, 2H), 2.73 (br s, 2H), 3.08 (s, 6H), 3.33 (t, *J* = 7.0 Hz, 4H), 3.42 (q, *J* = 7.0 Hz, 8H),, 3.93 (t, *J* = 6.0 Hz, 2H),

6.74 (d, J = 8.5 Hz, 4H), 6.99 (d, J = 8.5 Hz, 4H). ^{13}C NMR (126 MHz, $CDCl_3$) δ 8.49, 22.51, 25.86, 26.16, 28.37, 29.11, 34.58, 37.34, 39.76, 57.03, 61.00, 67.59, 114.06, 129.63, 130.73, 136.03, 146.09, 157.26. HRMS (ESI, M^{2+}) $C_{45}H_{72}O_2N_2$ Calcd. 336.2804, found 336.2797.

*6-(4-((E)-((5R,7R)-Adamantan-2-ylidene)(4-((6-azidohexyl)oxy)phenyl)methyl)phenoxy)hexyl methanesulfonate (**6**)*

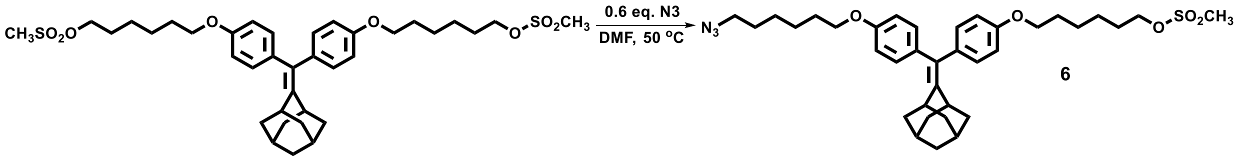

6

The mixture of the compound **2** (34 mg, 0.05 mmol) and NaN_3 (2.0 mg, 0.03 mmol) in DMF (100 µL) was stirred for 1 hr at 50 °C. The solvent was removed under a gentle stream of nitrogen, followed by dissolution of the residue into EtOAc (200 µL) to load a SiO_2 Preparative TLC (20 x 20 cm) that was developed with 10% EtOAc/n-Hexane and from which the title compound **6** (20 mg) (film type solid) was obtained.

^{1}H NMR (500 MHz, $CDCl_3$) δ 1.42-1.57 (m, 8H), 1.65 (quintet, J = 7.5 Hz, 2H), 1.76~1.92 (m, 14H), 2.01 (br s, 2H), 2.81 (br s, 2H), 3.01 (s, 3H), 3.30 (t, J = 7.0 Hz, 2H), 3.95 (t, J = 6.0 Hz, 4H), 4.26 (t, J = 7.0 Hz, 2H), 6.79 (d, J = 8.5 Hz, 2H), 6.80 (d, J = 8.5 Hz, 2H), 7.03 (d, J = 8.5 Hz, 4H). ^{13}C NMR (126 MHz, $CDCl_3$) δ 25.49, 25.87, 25.99, 26.77, 28.47, 29.06, 29.33, 29.38, 29.46, 34.67, 37.44, 37.62, 39.85, 51.65, 67.69, 67.78, 70.23, 113.98, 129.85, 130.84, 135.94, 135.99, 145.94, 157.44, 157.48. HRMS (ESI, M^{+} + 1) $C_{36}H_{50}O_5N_3S$ Calcd. 636.3471, found 636.3451.

*1-(6-(4-((Z)-((5r,7r)-adamantan-2-ylidene)(4-((6-azidohexyl)oxy)phenyl)methyl)phenoxy)hexyl)azepane (**N_3-NB-72**)*

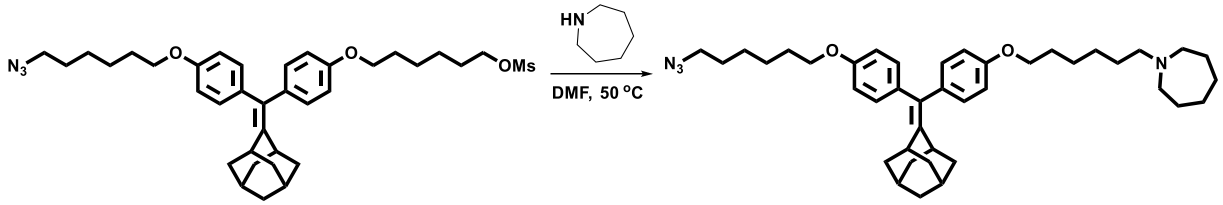

N_3-NB-72 (12 mg) was obtained from the reaction of the compound **6** (15 mg, 0.024 mmol) and azepane (20 mg, 0.02 mmol) following the procedure described for the preparation of **NB-65**.

^{1}H NMR (500 MHz, $CDCl_3$) δ 1.37 (quintet, J = 7.0 Hz, 2H), 1.44-1.57 (m, 8H), 1.59~1.70 (m, 10H), 1.76~1.84 (m, 4H), 1.84~1.93 (m, 10H), 2.02 (br s, 2H), 2.49 (t, J = 7.5 Hz, 2H), 2.64 (t, J = 6.0 Hz, 4H), 2.82 (br s, 2H), 3.31 (t, J = 7.0 Hz, 2H), 3.95 (q, J = 6.0 Hz, 4H), 6.81 (d, J = 8.5 Hz, 4H), 7.03 (d, J = 8.5 Hz, 2H), 7.04 (d, J = 8.5 Hz, 2H). ^{13}C NMR (126 MHz, $CDCl_3$) δ 25.99, 26.33, 27.26, 27.65, 27.67, 28.08, 28.48, 29.06, 29.46, 29.59, 34.67, 37.46, 39.86, 51.65, 55.82, 58.50, 67.77, 67.99, 113.98, 113.99, 129.90, 130.81, 130.84, 135.85, 135.97, 145.87, 157.46, 157.56. HRMS (ESI, M^+ + 1) $C_{41}H_{59}O_2N_4$ Calcd. 639.4638, found 639.4639.

*6-(4-((E)-((5r,7r)-adamantan-2-ylidene)(4-((6-(azepan-1-yl)hexyl)oxy)phenyl)methyl)phenoxy)hexan-1-amine (**NH_2-NB-72**)*

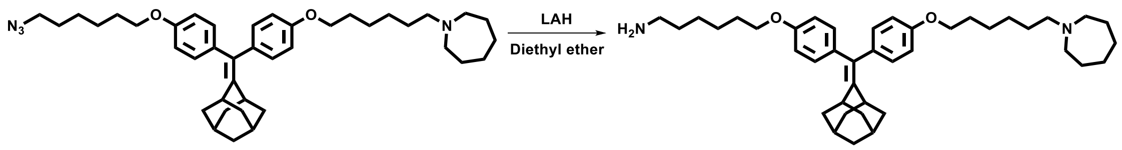

LAH (lithium aluminum hydride**,** 8 mg**,** 0.21 mmol) was added to the solution **N_3-NB-72** (10 mg, 0.016 mmol) in diethyl ether (500 µL), and the reaction was left in an inert atmosphere environment for 1 hr at rt. To this was added 10% NaOH aqueous solvent, and product was extracted with dichloromethane (200 µL x 4), washed with a deionized water, and dried over Na_2SO_4. Evaporation of solvent afforded **NH_2-NB-72** (5 mg) as a film type solid that was used without further purification.

^{1}H NMR (500 MHz, $CDCl_3$) δ 1.30~1.43 (m, 4H), 1.45~1.58 (m, 8H), 1.59~1.69 (m, 8H), 1.76~1.84 (m, 4H), 1.84~1.94 (m, 10H), 2.02 (br s, 2H), 2.48 (t, *J* = 7.5 Hz, 2H), 2.64 (t, *J* = 6.0 Hz, 4H), 2.71 (t, *J* = 7.5 Hz, 2H), 2.82 (br s, 2H), 3.95 (q, *J* = 6.0 Hz, 4H), 6.80 (d, *J* = 8.5 Hz, 4H), 7.04 (d, *J* = 8.5 Hz, 4H). ESI (m/z, $(M+2H)^{2+}$) 307.6.

*5-((6-(4-((E)-((5r,7r)-adamantan-2-ylidene)(4-((6-(azepan-1-yl)hexyl)oxy)phenyl)methyl)phenoxy)hexyl)carbamoyl)-2-(6-hydroxy-3-oxo-3H-xanthen-9-yl)benzoic acid (**Fluorescein-NB-72**)*

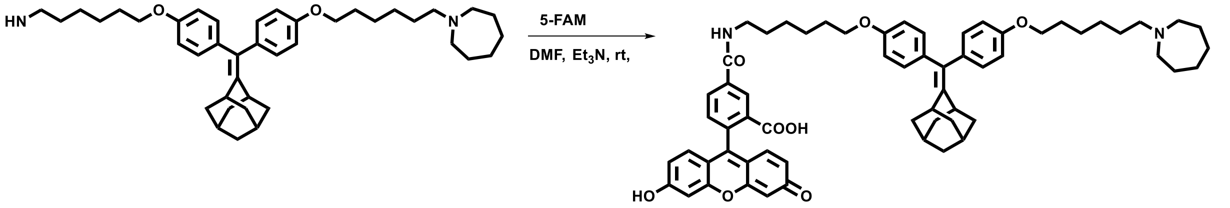

To the mixture of **NH_2-NB-72** (3 mg, 4.90 µmol) and 5-FAM (3.4 mg, 7.40 µmol) in DMF (100 µL) was added (11 mg, 0.11 mmol) at rt and reacted for 1 hr at rt. After the solvent was evaporated using a nitrogen stream, the title compound (**Fluorescein-NB-72,** 1.4 mg) was separated on SiO_2 PLC using 10% MeOH/DCM (v/v). ESI (m/z) 971.9 (M^+ +1), 487.6 (M/Z^{2+2H})

*1-(6-(4-((Z)-((5r,7r)-adamantan-2-ylidene)(4-((6-azidohexyl)oxy)phenyl)methyl)phenoxy)hexyl)-1-methyl-1λ^4-azepane (**N_3-NB-73**)*

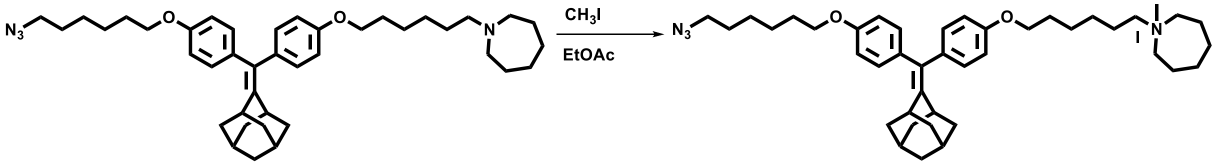

N_3-NB-73 (3.7 mg) was obtained from the reaction of the **N_3-NB-72** (3 mg, 4.89 µmol) and methyl iodide (100 µL) in ethyl acetate solvent (100 µL) following the procedure described for the preparation of **NB-68**.

^{1}H NMR (500 MHz, $CDCl_3$) δ 1.49-1.51 (m, 8H), 1.65 (quintet, *J* = 7.0 Hz, 2H), 1.72~1.92 (m, 20H), 1.92~2.05 (m, 6H), 2.79 (br s, 2H), 3.29 (t, *J* = 7.0 Hz, 2H), 3.33 (s, 3H), 3.60~3.70 (m, 6H), 3.94 (t, *J* = 6.0 Hz, 2H), 3.95 (t, *J* = 6.0 Hz, 2H), 6.80 (d, *J* = 8.5 Hz, 4H), 7.03 (d, *J* = 8.5 Hz, 2H), 7.04 (d, *J* = 8.5 Hz, 2H). ^{13}C NMR (126 MHz, $CDCl_3$) δ 22.39, 23.14, 25.98, 26.02, 26.25, 26.76, 27.64, 28.46, 29.05, 29.28, 29.46, 34.67, 37.44, 39.86, 51.64, 65.21, 65.30, 67.63, 67.83, 114.03, 114.06, 129.82, 130.79, 130.82, 135.95, 136.02, 146.03, 157.40, 157.50. HRMS (ESI, M^+) $C_{42}H_{61}O_2N_4$ Calcd. 653.4795, found 653.4780.

*5-(((1-(6-(4-((Z)-((5r,7r)-adamantan-2-ylidene)(4-((6-(1-methyl-1λ^4-azepan-1-yl)hexyl)oxy)phenyl)methyl)phenoxy)hexyl)-1H-1,2,3-triazol-4-yl)methyl)carbamoyl)-2-(6-hydroxy-3-oxo-3H-xanthen-9-yl)benzoic acid iodide (**Fl-NB-73**)*

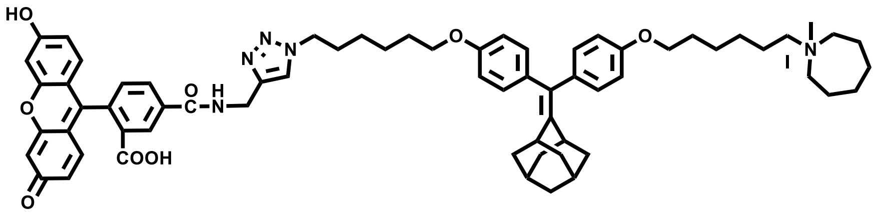

The mixture of the **N_3-NB-73** (3 mg, 3.8 µmol) and fluorescein-propargyl amide (3 mg, 7.2 µmol) in the presence of 0.2 mol% CuI in a 1:1 mixture of DIEA:AcOH (20 µL) in DCM (200 µL) was stirred for 2 hr at rt. The reaction mixture was loaded directly onto a SiO_2 TLC plate (20 x 20 cm). Development with 25% MeOH/DCM (v/v) provided the title compound **Fl-NB-73** (2.1 mg, Rf = 0.15).

HRMS (ESI, M^+) $C_{66}H_{76}O_8N_5$ Calcd. 1066.5694, found 1066.5677.
